# Circumstances of fall-related injuries by age and gender among community-dwelling adults in the United States

**DOI:** 10.1371/journal.pone.0176561

**Published:** 2017-05-04

**Authors:** Lava R. Timsina, Joanna L. Willetts, Melanye J. Brennan, Helen Marucci-Wellman, David A. Lombardi, Theodore K. Courtney, Santosh K. Verma

**Affiliations:** 1 Center for Injury Epidemiology, Liberty Mutual Research Institute for Safety, Hopkinton, Massachusetts, United States of America; 2 College of Public Health, University of Kentucky, Lexington, Kentucky, United States of America; 3 Environmental and Occupational Medicine and Epidemiology Program, Department of Environmental Health, Harvard T.H. Chan School of Public Health, Boston, Massachusetts, United States of America; 4 Department of Family Medicine and Community Health, University of Massachusetts Medical School, Worcester, Massachusetts, United States of America; Northwestern University, UNITED STATES

## Abstract

**Introduction:**

Falls are the leading cause of injury in almost all age-strata in the U.S. However, fall-related injuries (FI) and their circumstances are under-studied at the population level, particularly among young and middle-aged adults. This study examined the circumstances of FI among community-dwelling U.S. adults, by age and gender.

**Methods:**

Narrative texts of FI from the National Health Interview Survey (1997–2010) were coded using a customized taxonomy to assess place, activity, initiating event, hazards, contributing factors, fall height, and work-relatedness of FI. Weighted proportions and incidence rates of FI were calculated across six age-gender groups (18–44, 45–64, 65+ years; women, men).

**Results:**

The proportion of FI occurring indoors increased with age in both genders (22%, 30%, and 48% among men, and 40%, 49% and 62% among women for 18–44, 45–64, 65+ age-groups, respectively). In each age group the proportion of indoor FI was higher among women as compared to men. Among women, using the stairs was the second leading activity (after walking) at the time of FI (19%, 14% and 10% for women in 18–44, 45–64, 65+ age groups, respectively). FI associated with tripping increased with age among both genders, and women were more likely to trip than men in every age group. Of all age-gender groups, the rate of FI while using ladders was the highest among middle-aged men (3.3 per 1000 person-year, 95% CI 2.0, 4.5). Large objects, stairs and steps, and surface contamination were the three most common hazards noted for 15%, 14% and 13% of fall-related injuries, respectively.

**Conclusions:**

The rate and the circumstances of FI differ by age and gender. Understanding these differences and obtaining information about circumstances could be vital for developing effective interventions to prevent falls and FI.

## Introduction

In the United States, falls are the leading cause of medically attended nonfatal injuries,[1] and fall-related injuries (FI) have been increasing in recent years.[2–7] A few studies have noted that the increasing trend in FI incidence exceeds what would be expected due to the aging of the population in the U.S.[8–11] Therefore, greater understanding of the circumstances and mechanisms of falls is needed in order to better address these concerning trends.

FI can be reduced by addressing both the intrinsic risk factors for falls such as polypharmacy, balance, vision problems, etc.,[12] and also by designing targeted fall prevention interventions based on the understanding of circumstances surrounding FI events, such as the place, activity, initiating events, and hazards, that may predispose or precipitate FI.[13] A study of fractures found that outdoor falls among adults aged 45 years and older were frequently attributed to modifiable environmental factors that were extrinsic in nature.[14] Identifying specific circumstances of FI may help in prioritizing new areas within falls research and develop targeted intervention programs.

In addition, the circumstances of FI may differ by age and gender. A longitudinal study, based in the Baltimore-Washington DC area, reported significant differences between young (20–45 years), middle-aged (46–65 years) and older adults (> 65 years) with respect to activities leading to falls.[15] The MOBILIZE Boston cohort study found that among adults over 65 years of age, women’s overall rate of injurious indoor falls were nearly twice that of men’s, and the injurious outdoor fall rates were equivalent in both sexes.[16] Kelsey et al. found that older participants with poor baseline health characteristics had elevated rates of indoor falls while transitioning, walking, or not moving, and healthy older adults had elevated rates of outdoor falls during walking and vigorous activity.[17] They concluded that fall prevention programs should be tailored to personal characteristics, activities, and locations.

Most previous studies have examined circumstances of FI among older adults and/or in limited geographical areas with small sample sizes. [14, 16, 18,19] Few studies have examined the circumstances of FI at the national level and across the adult life span, particularly among young and middle-aged adults. Despite being the leading cause of nonfatal injuries among young and middle-aged adults, falls remain an under-studied public health problem in these populations. [11, 15, 20–22] The primary objective of this study was to utilize the considerable information in the National Health Interview Survey (NHIS) injury narratives to examine the circumstances of medically attended FI among community-dwelling (the non-institutionalized civilian) U.S. adults. We have provided both the proportions and incidence rates of FI related to different circumstances for the overall adult population and the six age-gender groups. Proportions highlight the target intervention areas within each age-gender group and incidence rates are useful in understanding how a circumstance of fall affects the risk of FI across the age-gender groups.

## Materials and methods

### Data and study design

The NHIS is a population-based survey conducted by the National Center for Health Statistics (NCHS) to collect information about health, socioeconomic and demographic factors. [23] It is designed to produce national estimates for the non-institutionalized civilian U.S. population. The NHIS surveys one sample adult (18+) from each household on more detailed health and lifestyle topics.

The NHIS collects information on all injuries requiring medical attention to any family member during the three months prior to the interview. [24–27] Injury information is collected for all members of the family in response to the screening questions about getting injured and seeking medical care or advice. In this study, the analysis was restricted to the sample adult core (who would respond for themselves) to avoid proxy responses. The injury narrative verbatim responses, and the NHIS coded variables describing where the injury occurred and the activity at the time of injury, were used to code circumstances of FI. The average length of injury narrative was 64 characters (Range 1–255; For example—WHILE TAKING OUT GARBAGE HE SLIPPED ON THE ICY STEP AND FELL).

Injuries in the NHIS are coded using the Ninth Revision of the International Classification of Disease external cause codes (ICD-9-CM). FI were identified using ICD-9-CM codes: E880-E888, “Accidental Falls.” Data from the NHIS, 1997–2010, were extracted and analyzed. This study utilized existing publically available NHIS data and was exempted from Institutional Review Board approval by New England Institutional Review Board.

### Narrative coding approach

Based on the approach proposed by Lincoln et al., [28] and the investigators’ prior methods of narrative text analysis, [29–31] a coding taxonomy was developed (S1 Table).

The coding taxonomy included eight categories to describe circumstances of FI:

1. *Place*: Location of FI.
2. *Activity*: Activity that the respondent (injured person) was performing when the FI occurred. Walking was the default category if the narrative indicated slips or trips as initiating events with no other information; otherwise, “‘Other/Unknown” was the default.
3. *Initiating event*: Event that initiated the fall.
4. *Hazards*: Extrinsic factors that may have been directly related to FI. For example, “Contaminants on the surface,” “Objects on the floor,” etc. We allowed multiple hazard codes for each FI.
5. *Level*: Identified whether the FI occurred from a same-level or to a lower level fall. Same-level fall was defined as a fall at the level (or higher) the person was standing (Please see S1 Table for detail). Same-level was the default category when no additional information was available.
6. *Work-relatedness*: Identified whether a person was working at a paid job when the FI occurred.
7. *Contributing factors*: Results are not shown (82% of the narratives did not have sufficient information).
8. *Direction of fall*: Results are not shown (82% of the narratives did not have sufficient information).

### Coder training and testing

Two coders were initially trained on a random sample of 200 FI narratives. Each coder was then provided with another 200 injury narratives, which were independently coded. The Kappa scores, used to assess inter-rater reliability of the coded circumstances, ranged from 0.98 to 1.00. Subsequently, narratives for circumstances in which two categories were similar were reviewed for coding consistency. For example, if the fall hazard was identified to be a ladder, then narratives were reviewed to determine whether the activity should be defined as working from a ladder and vice versa. Out of 30,091 codes assigned to 8 categories, 40 codes (0.13%) were reassigned after these consistency checks.

### Statistical analysis

Six age-gender groups were considered in this study: 18–44 years (young adults), 45–64 years (middle-aged adults), and 65+ years (older adults); women and men. Proportions (number of FI related to a particular circumstance within each category ÷ total number of FI) and incidence rates (IR, number of FI related to a particular circumstance within each category ÷ total number of sampled adults) of FI related to a particular circumstance were tabulated overall and for the six age-gender groups. For the incidence rate of FI, the three-month estimates were multiplied by 4 to create annual estimates of FI for each year, and then an average of the annual estimates from data pooled over 14 years (1997–2010) was calculated. The incidence rates calculated in the study use the estimated within-group adult population for the denominator (IR per 1000 population) and do not account for the participants’ actual time of exposure to particular circumstances. Weighted statistics were obtained using complex survey procedures in SAS 9.3.[32] Results are not shown when the unweighted number of injuries for a circumstance was ten or less for the overall sample, or five or less for each age-gender group.

## Results

Of the 414,044 sample adults interviewed over the 14-year period (1997–2010), a total of 4,018 reported at least one FI in the past three months. Of these, 204 (5%) reported more than one FI. When weighed, this represents an annual estimate of 1.9 million adults reporting a FI in the previous three months, out of 213 million community-dwelling U.S. adults. The demographic characteristics representing U.S. adults and adults who reported at least one FI in the previous three months are presented in Table 1. Eighty-seven out of 4222 narratives (2.1%) did not provide any information for any of the eight categories.

**Table 1:** Demographic characteristics of sample adults (weighted average per year N = 212,507,916) and adults reporting medically attended fall-related injuries in the previous three months (weighted average per year n = 1,904,985).

Variables	Sample Adults		Adult with a Fall-related Injury	
	n (000)	% (95% CI)	n (000)	% (95% CI)
Overall	212,508	100.0	1,905	100.0
**Gender and Age Group**				
**Males**	**102,240**	**48.1 (47.9, 48.3)**	**727**	**38.2 (36.4, 39.9)**
18–44 years	54,348	25.6 (25.3, 25.8)	340	17.8 (16.3, 19.4)
45–64 years	33,104	15.6 (15.4, 15.7)	224	11.8 (10.6, 13)
65+ years	14,788	7.0 (6.8, 7.1)	163	8.5 (7.4, 9.7)
**Females**	**110,268**	**51.9 (51.7, 52.1)**	**1,178**	**61.8 (60.1, 63.6)**
18–44 years	55,264	26 (25.8, 26.2)	374	19.6 (18.2, 21.0)
45–64 years	35,159	16.5 (16.4, 16.7)	398	20.9 (19.5, 22.3)
65+ years	19,844	9.3 (9.2, 9.5)	406	21.3 (19.9, 22.7)
**Race/Ethnicity**				
Non-Hispanic White	152,509	71.8 (71.3, 72.2)	1,557	81.8 (80.5, 83.1)
Non-Hispanic Black	24,370	11.5 (11.1, 11.8)	154	8.1 (7.2, 8.9)
Hispanic	25,643	12.1 (11.7, 12.4)	132	6.9 (6.1, 7.7)
Non-Hispanic Other	9,986	4.7 (4.5, 4.9)	62	3.3 (2.5, 4)
**Educational attainment**				
Did not complete high school	35,196	16.6 (16.3, 16.8)	333	17.5 (16.1, 18.8)
High School Graduate	61,489	28.9 (28.6, 29.2)	530	27.8 (26.2, 29.5)
Some college and beyond	113,765	53.5 (53.1, 54)	1,033	54.2 (52.3, 56.1)
No information	2,058	1 (0.9, 1)	9	0.5 (0.3, 0.7)

### Place

Among community-dwelling adults, slightly more FI occurred outdoors (47%) than indoors (43%) (Table 2); however, the distribution of outdoor and indoor FI varied by age and gender. Among young men, the proportion of outdoor FI was more than twice the proportion of indoor FI (62.8% and 22.3%, respectively), whereas among older women, 32.8% of FI occurred outdoors and 61.9% occurred indoors.

**Table 2:** Place of fall-related injuries (proportion* (%) and incidence rate (IR) per 1000 person-years and their 95% confidence intervals) by age and gender.

PLACE	Overall			18–44 years				45–64 years				65+ years			
				Males (SA = 54,348 FI = 1,396)^a^		Females (SA = 55,264 FI = 1,576)^a^		Males (SA = 33,104 FI = 972)^a^		Females (SA = 35,159 FI = 1,666)^a^		Males (SA = 14,788 FI = 679)^a^		Females (SA = 19,844FI = 1,706)^a^	
	FI (000)	% (95% CI)	IR (95% CI)	%(95% CI)	IR(95% CI)	%(95% CI)	IR(95% CI)	% (95% CI)	IR(95% CI)	%(95% CI)	IR (95% CI)	%(95% CI)	IR (95% CI)	% (95% CI)	IR(95% CI)
**INDOOR**	3,434	42.9 (41.0, 44.8)	16.2 (15.1, 17.2)	22.3 (18.3, 26.2)	5.7 (4.6, 6.9)	39.8 (35.6, 43.9)	11.3 (9.7, 13.0)	30.2 (24.7, 35.7)	8.9 (6.9, 10.9)	49.3 (45.7, 53.0)	23.4 (20.6, 26.2)	47.6 (41.8, 53.4)	21.9 (17.5, 26.2)	61.9 (58.4, 65.5)	53.3 (47.8, 58.7)
**Home**	2,735	34.2 (32.3, 36.1)	12.9 (11.9, 13.8)	13.2 (10.1, 16.3)	3.4 (2.5, 4.3)	30.0 (26.0, 34.1)	8.6 (7.1, 10.1)	24.7 (19.2, 30.1)	7.3 (5.3, 9.2)	37.1 (33.5, 40.8)	17.6 (15.3, 19.9)	41.0 (35.0, 46.9)	18.8 (14.8, 22.8)	55.1 (51.4, 58.8)	47.4 (42.3, 52.4)
**Bedroom**	220	2.8 (2.2, 3.3)	1.0 (0.8, 1.3)	-	-	1.2 (0.3, 2.1)	0.4 (0.1, 0.6)	2.3^u^ (0.5, 4.0)	0.7 ^u^ (0.2, 1.2)	2.3 (1.3, 3.3)	1.1 (0.6, 1.6)	4.8 (2.4, 7.3)	2.2 (1.0, 3.4)	5.7 (4.3, 7.2)	4.9 (3.6, 6.3)
**Kitchen**	162	2.0 (1.6, 2.5)	0.8 (0.6, 0.9)	-	-	1.9 (0.9, 2.8)	0.5 (0.3, 0.8)	-	-	2.0 (1.0, 2.9)	0.9 (0.5, 1.4)	2.5 (0.9, 4.1)	1.2 (0.4, 1.9)	4.1 (2.7, 5.4)	3.5 (2.3, 4.7)
**Restroom/Bathroom**	273	3.4 (2.8, 4.1)	1.3 (1.0, 1.5)	1.1 ^u^ (0.2, 2.0)	0.3 ^u^ (0.1, 0.5)	2.4 (1.1, 3.7)	0.7 (0.3, 1.1)	1.0 ^u^ (0.2, 1.9)	0.3 ^u^ (0.1, 0.6)	4.6 (2.8, 6.4)	2.2 (1.3, 3.1)	5.0 (2.5, 7.5)	2.3 (1.1, 3.5)	5.8 (4.3, 7.3)	5.0 (3.6, 6.4)
**Stairs/Steps**	455	5.7 (4.8, 6.5)	2.1 (1.8, 2.5)	2.7 (1.4, 3.9)	0.7 (0.3, 1.0)	9.0 (6.3, 11.8)	2.6 (1.8, 3.4)	3.9 (2.1, 5.6)	1.1 (0.6, 1.7)	6.8 (4.9, 8.8)	3.2 (2.3, 4.2)	2.8 (1.2, 4.3)	1.3 (0.5, 2.1)	6.1 (4.3, 7.9)	5.3 (3.5, 7.0)
**Non-Home**	699	8.7 (7.7, 9.8)	3.3 (2.9, 3.7)	9.1 (6.4, 11.8)	2.3 (1.6, 3.0)	9.7 (7.1, 12.3)	2.8 (2.0, 3.6)	5.6 (3.3, 7.8)	1.6 (0.9, 2.4)	12.2 (9.7, 14.7)	5.8 (4.3, 7.3)	6.7 (3.4, 9.9)	3.1 (1.5, 4.7)	6.9 (5.0, 8.7)	5.9 (4.2, 7.6)
**Public Restroom or Bathroom**	36	0.4 (0.2, 0.7)	0.2 (0.1, 0.3)	-	-	-	-	-	-	-	-	-	-	-	-
**Sporting Facility**	46	0.6 (0.3, 0.9)	0.2 (0.1, 0.3)	-	-	1.1 ^u^ (0.3, 1.9)	0.3 ^u^ (0.1, 0.6)	-	-	-	-	-	-	-	-
**OUTDOOR**	3,756	47.0 (45.1, 48.9)	17.7 (16.6, 18.7)	62.8 (58.2, 67.4)	16.1 (14.1, 18.1)	48.6 (44.3, 52.9)	13.9 (12.2, 15.6)	51.7 (45.7, 57.7)	15.2 (12.6, 17.8)	43.5 (40.0, 47.0)	20.6 (18.4, 22.8)	48.1 (42.5, 53.7)	22.1 (18.2, 26.0)	32.8 (29.4, 36.2)	28.2 (24.6, 31.8)
**Parking Lot**	278	3.5 (2.8, 4.1)	1.3 (1.1, 1.6)	3.0 (1.4, 4.7)	0.8 (0.3, 1.2)	4.4 (2.9, 5.8)	1.2 (0.8, 1.7)	3.5 (1.8, 5.1)	1.0 (0.5, 1.5)	4.7 (3.2, 6.3)	2.3 (1.5, 3.0)	1.9 ^u^ (0.4, 3.4)	0.9 ^u^ (0.2, 1.5)	2.4 (1.4, 3.4)	2.1 (1.2, 3.0)
**Sidewalk/Curb**	310	3.9 (3.2, 4.6)	1.5 (1.2, 1.7)	1.7 (0.6, 2.8)	0.4 (0.2, 0.7)	4.3 (2.8, 5.9)	1.2 (0.8, 1.7)	2.2 (0.9, 3.4)	0.6 (0.3, 1.0)	4.2 (2.5, 5.9)	2.0 (1.2, 2.8)	6.6 (3.6, 9.7)	3.0 (1.6, 4.5)	4.8 (3.2, 6.4)	4.1 (2.6, 5.7)
**Sidewalk**	199	2.5 (1.9, 3.1)	0.9 (0.7, 1.2)	-	-	2.5 (1.2, 3.7)	0.7 (0.4, 1.1)	1.4 ^u^ (0.4, 2.5)	0.4 ^u^ (0.1, 0.7)	3.0 (1.5, 4.5)	1.4 (0.7, 2.2)	4.3 (1.9, 6.7)	2.0 (0.9, 3.1)	3.4 (1.9, 4.9)	2.9 (1.6, 4.3)
**Curb**	111	1.4 (1.0, 1.8)	0.5 (0.4, 0.7)	1.1 ^u^ (0.3, 1.8)	0.3 ^u^ (0.1, 0.5)	1.9 (0.9, 2.8)	0.5 (0.3, 0.8)	0.8 ^u^ (0.1, 1.4)	0.2 ^u^ (0.0, 0.4)	1.2 (0.5, 1.9)	0.6 (0.2, 0.9)	2.3 ^u^ (0.3, 4.4)	1.1 ^u^ (0.1, 2.0)	1.4 (0.6, 2.2)	1.2 (0.5, 1.9)
**Street**	208	2.6 (2.1, 3.1)	1.0 (0.8, 1.2)	2.3 (1.2, 3.5)	0.6 (0.3, 0.9)	1.5 (0.6, 2.4)	0.4 (0.2, 0.7)	3.7 (1.6, 5.8)	1.1 (0.4, 1.7)	3.0 (2.1, 4.0)	1.4 (0.9, 2.0)	2.1 ^u^ (0.8, 3.5)	1.0 ^u^ (0.3, 1.6)	3.0 (1.9, 4.1)	2.6 (1.6, 3.5)
**Park/Recreation Area/ Sporting Facility**	771	9.6 (8.5, 10.7)	3.6 (3.2, 4.1)	26.2 (22.2, 30.2)	6.7 (5.5, 8.0)	12.1 (9.2, 14.9)	3.4 (2.6, 4.3)	10.5 (6.7, 14.3)	3.1 (1.9, 4.3)	3.9 (2.3, 5.4)	1.8 (1.1, 2.6)	3.1 (1.4, 4.8)	1.4 (0.6, 2.3)	1.6 (0.8, 2.4)	1.4 (0.7, 2.1)
**Home**	1,766	22.1 (20.6, 23.6)	8.3 (7.6, 9.0)	19.3 (15.7, 22.9)	5.0 (3.9, 6.0)	22.1 (18.6, 25.5)	6.3 (5.1, 7.4)	26.8 (21.6, 32.0)	7.9 (5.9, 9.9)	22.7 (19.6, 25.8)	10.8 (9.0, 12.5)	30.9 (25.5, 36.2)	14.2 (11.0, 17.4)	17.6 (15.1, 20.1)	15.1 (12.7, 17.6)
**Lawn/Yard/Garden**	352	4.4 (3.7, 5.1)	1.7 (1.4, 1.9)	1.3 (0.5, 2.0)	0.3 (0.1, 0.5)	4.1 (2.6, 5.5)	1.2 (0.7, 1.6)	3.9 (2.0, 5.8)	1.2 (0.5, 1.9)	5.2 (3.6, 6.8)	2.5 (1.7, 3.2)	8.4 (5.3, 11.6)	3.9 (2.2, 5.5)	5.2 (3.6, 6.8)	4.5 (3.0, 5.9)
**Driveway/Parking Space/Sidewalk/ Pavement**	116	1.4 (1.1, 1.8)	0.6 (0.4, 0.7)	-	-	1.6 (0.6, 2.5)	0.4 (0.2, 0.7)	1.7 ^u^ (0.5, 2.9)	0.5 ^u^ (0.2, 0.9)	1.5 (0.8, 2.2)	0.7 (0.3, 1.1)	2.1 ^u^ (0.5, 3.7)	1.0 ^u^ (0.2, 1.7)	1.8 (1.0, 2.6)	1.6 (0.9, 2.3)
**Porch/Deck/Pools**	213	2.7 (2.1, 3.2)	1.0 (0.8, 1.2)	3.0 (1.2, 4.8)	0.8 (0.3, 1.2)	1.9 (0.9, 3.0)	0.6 (0.2, 0.9)	2.0 ^u^ (0.5, 3.5)	0.6 ^u^ (0.2, 1.0)	3.1 (1.9, 4.4)	1.5 (0.9, 2.1)	4.1 (1.8, 6.4)	1.9 (0.8, 3.0)	2.4 (1.4, 3.4)	2.1 (1.2, 3.0)
**Stairs/Steps**	231	2.9 (2.3, 3.4)	1.1 (0.9, 1.3)	1.7 (0.8, 2.6)	0.4 (0.2, 0.7)	4.1 (2.6, 5.6)	1.2 (0.7, 1.6)	1.9 ^u^ (0.4, 3.4)	0.6 ^u^ (0.1, 1.0)	3.1 (1.9, 4.2)	1.5 (0.9, 2.0)	4.4 (1.5, 7.2)	2.0 (0.6, 3.4)	2.5 (1.5, 3.5)	2.2 (1.3, 3.0)
**PLACE NOT SPECIFIED OR UNKNOWN**	804	10.1 (8.9, 11.2)	3.8 (3.3, 4.3)	14.9 (11.3, 18.4)	3.8 (2.8, 4.9)	11.6 (8.9, 14.2)	3.3 (2.5, 4.1)	18.2 (13.2, 23.3)	5.4 (3.6, 7.1)	7.1 (5.1, 9.0)	3.4 (2.4, 4.3)	4.3 (1.7, 7.0)	2.0 (0.7, 3.3)	5.3 (3.7, 6.8)	4.5 (3.2, 5.9)

^a^ in thousands, SA = weighted Sampled Adults per year, FI = weighted average fall-related injuries per year, IR = Incidence Rate Per 1000 person-years, CI = Confidence Interval

* Proportions may not add up to 100% as cells with unweighted count of 10 or less for the overall category and 5 or less for age-gender groups are not shown

^u^ unweighted count between 6–10, the estimates may be unstable

**Fig 1:**
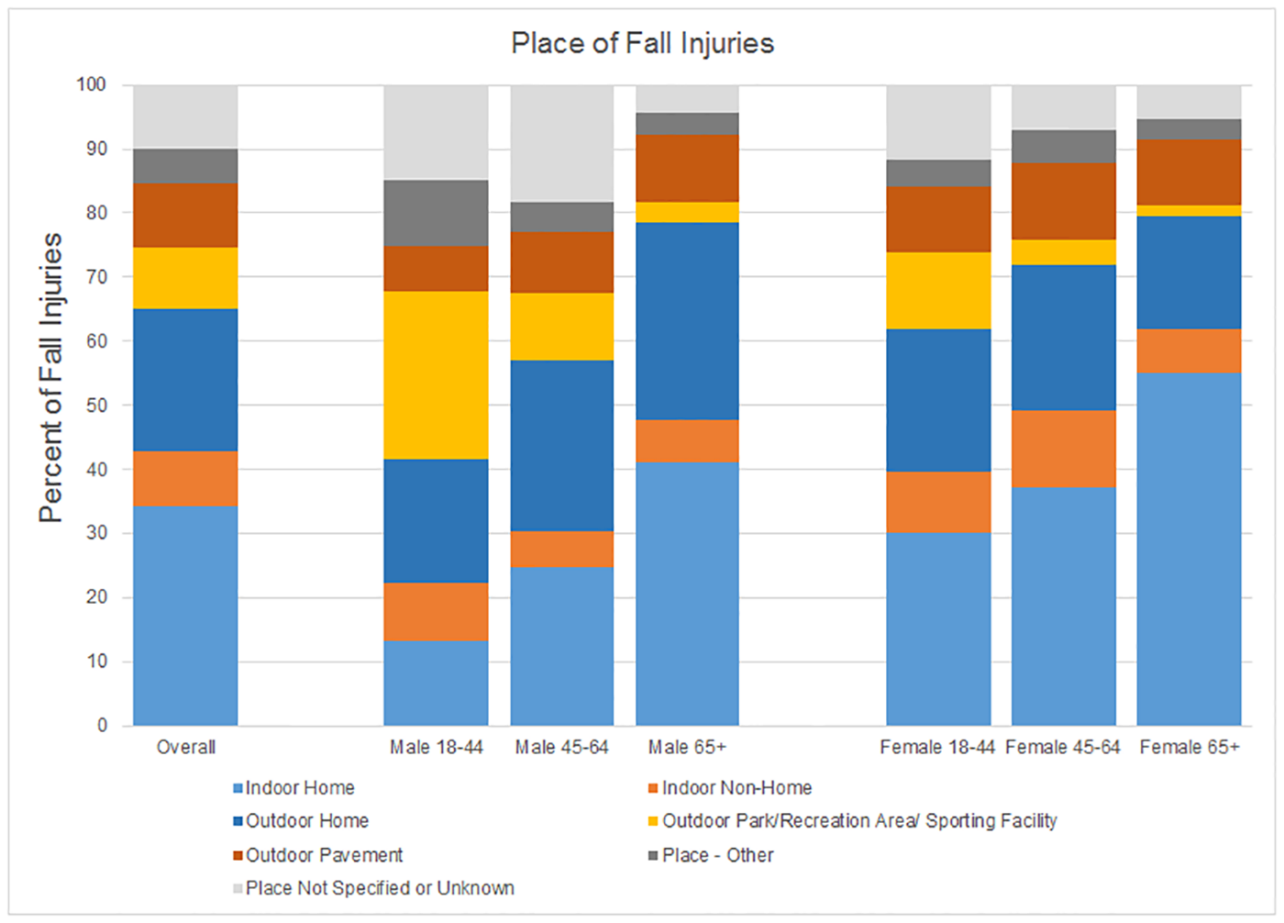
Place of fall injuries (%). Outdoor pavement included parking lots, sidewalks, curbs and streets.

About 2.7 million FI occurred annually inside the home and 1.8 million outside the home. The proportion of FI in and around the home was 56% (34.2% indoors, 22.1% outdoors) and this proportion was higher for older adults as compared to young adults. About 10% of all FI occurred on parking lots, sidewalks, curbs and streets (3.5%, 2.5%, 1.4%, and 2.6%, respectively; Fig 1.

The rate of indoor FI among older women was the highest among all age-gender groups (IR 53.3 FI per 1000 person-years, 95% CI 47.8, 58.7). However, the rate of outdoor FI was similar among older women, older men, and middle-aged women, and was higher than the rates in young men, young women and middle-aged men. The rate of FI at outdoor parks/recreation areas/sports facilities was the highest among young men (IR 6.7 per 1000 person-years, 95% CI 5.5, 8.0).

### Activity

**Fig 2:**
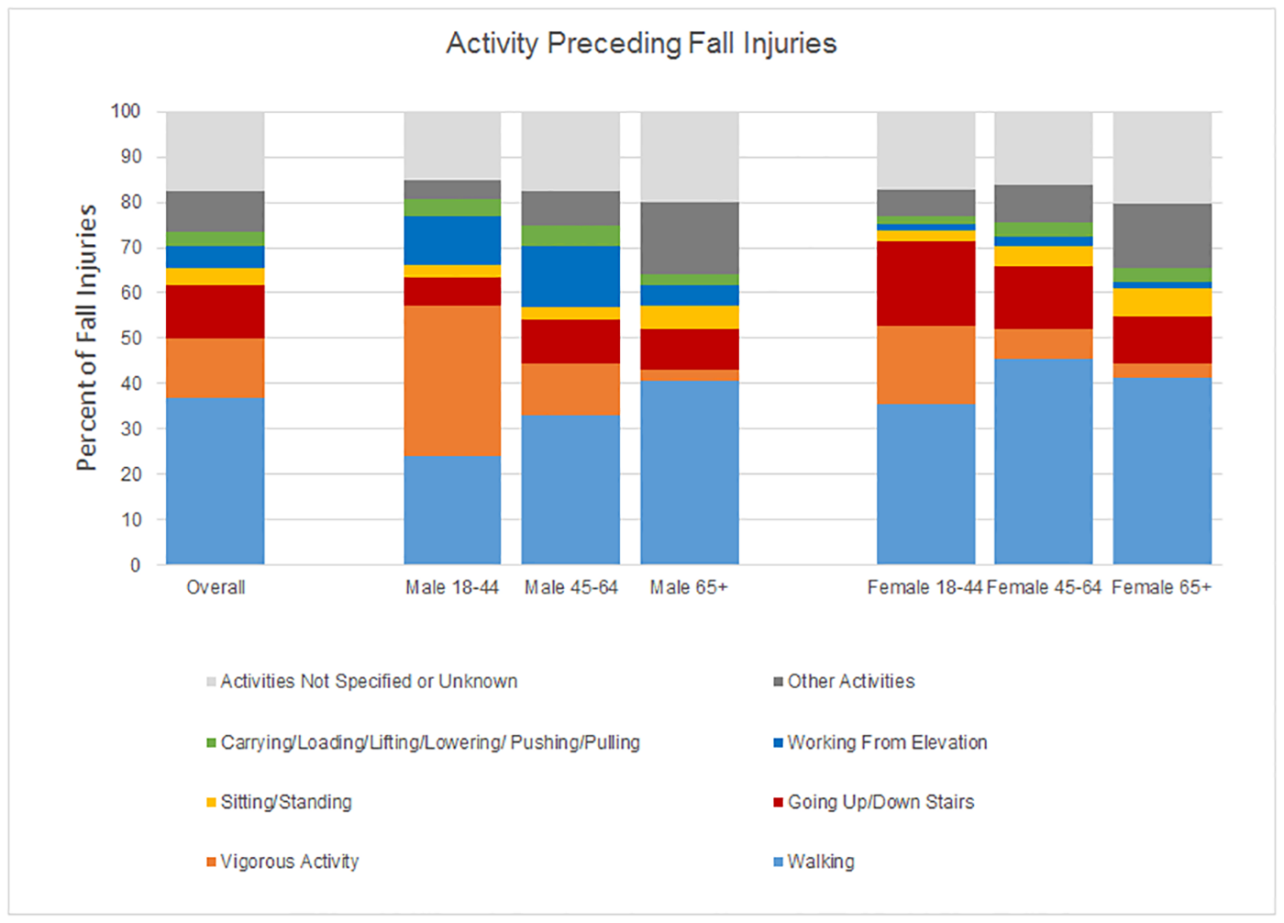
Activity preceding fall injuries (%). The proportion of FI while using stairs was the highest among young women (18.9%, 95% CI 15.1, 22.6), followed by middle-aged women (13.8%, 95% CI 11.2, 16.4) and older women (10.2%, 95% CI 8.1, 12.4). The proportion was less than 10% for men in all three age groups. The rate of FI while working on ladders was higher among young and middle-aged men as compared to women of the same age groups. An estimated 258 thousand FI occurred annually while on ladders among community-dwelling adults in the U.S.

Walking was the predominant activity preceding approximately 3 million FI annually (IR = 13.9 per 1000 person-years; 95% CI 13.0–14.9, 37% of all FI, Table 3). Vigorous activities, such as fast walking, running, playing sports, or exercising, (1.03 million FI annually) followed by going up or down the stairs (937 thousand FI annually) were other common activities being performed when FI occurred. Walking was the leading activity at the time of fall-related injuries for all age and gender groups (Fig 2) except young men who reported the highest prevalence of fall-related injuries while engaged in vigorous activity. For both genders, as age increased, the incidence rates of FI while “Playing/Sports/Exercising” decreased (Table 3).

**Table 3:** Activity preceding fall-related injuries (proportion* (%) and incidence rate (IR) per 1000 person-years and their 95% confidence intervals) by age and gender.

ACTIVITY	Overall			18–44 years				45–64 years				65+ years			
				Males SA = 54,348 FI = 1,396)^a^		Females (SA = 55,264 FI = 1,576)^a^		Males (SA = 33,104 FI = 972)^a^		Females (SA = 35,159 FI = 1,666)^a^		Males (SA = 14,788 FI = 679)^a^		Females (SA = 19,844 FI = 1,706)^a^	
	FI (000)	% (95% CI)	IR (95% CI)	% (95% CI)	IR (95% CI)	% (95% CI)	IR (95% CI)	% (95% CI)	IR (95% CI)	% (95% CI)	IR (95% CI)	% (95% CI)	IR (95% CI)	% (95% CI)	IR (95% CI)
**Moving Between Sitting/Standing Positions**	265	3.3 (2.6, 4.0)	1.3 (1.0, 1.5)	-	-	1.7 (0.8, 2.7)	0.5 (0.2, 0.8)	2.6 (0.5, 4.7)	0.8 (0.1, 1.4)	2.3 ^u^ (1.3, 3.3)	1.1 ^u^ (0.6, 1.6)	4.6 (2.0, 7.3)	2.1 (0.9, 3.4)	8.0 (5.8, 10.2)	6.9 (5.0, 8.8)
**Sitting/Standing**	321	4.0 (3.2, 4.8)	1.5 (1.2, 1.8)	2.7 (1.1, 4.2)	0.7 (0.3, 1.1)	2.4 (1.3, 3.6)	0.7 (0.4, 1.0)	2.9 (1.2, 4.7)	0.9 (0.4, 1.4)	4.3 (2.6, 6.1)	2.1 (1.2, 2.9)	5.2 (2.5, 7.9)	2.4 (1.0, 3.8)	6.4 (4.5, 8.2)	5.5 (3.8, 7.1)
**Standing**	259	3.2 (2.5, 4.0)	1.2 (0.9, 1.5)	2.3 (0.9, 3.7)	0.6 (0.2, 1.0)	2.2 (0.9, 3.5)	0.6 (0.3, 1.0)	2.2 (0.7, 3.8)	0.7 (0.2, 1.1)	2.9 (1.6, 4.2)	1.4 (0.7, 2.0)	4.6 (2.0, 7.2)	2.1 (0.8, 3.4)	5.3 (3.6, 7.0)	4.6 (3.0, 6.1)
**Sitting**	61	0.8 (0.4, 1.1)	0.3 (0.2, 0.4)	-	-	-	-	-	-	1.5 ^u^ (0.3, 2.6)	0.7 ^u^ (0.1, 1.3)	-	-	1.1 (0.5, 1.6)	0.9 (0.4, 1.4)
**Walking**	2,959	37.0 (35.3, 38.7)	13.9 (13.0, 14.9)	24.1 (20.0, 28.2)	6.2 (5.0, 7.4)	35.4 (31.5, 39.2)	10.1 (8.8, 11.4)	33.2 (27.6, 38.8)	9.8 (7.5, 12.1)	45.5 (41.8, 49.2)	21.6 (19.1, 24.1)	40.5 (35.1, 45.9)	18.6 (15.0, 22.2)	41.5 (37.7, 45.4)	35.7 (31.0, 40.4)
**Vigorous Activity**	1,029	12.9 (11.6, 14.2)	4.8 (4.3, 5.4)	33.3 (28.8, 37.9)	8.6 (7.1, 10.1)	17.2 (14.0, 20.4)	4.9 (3.9, 5.9)	11.4 (7.4, 15.4)	3.3 (2.1, 4.6)	6.7 (4.8, 8.5)	3.2 (2.1, 4.2)	2.7 (1.1, 4.4)	1.3 (0.4, 2.1)	3.1 (1.9, 4.3)	2.7 (1.7, 3.7)
**Walking Fast, Rushing, Running**	183	2.3 (1.7, 2.8)	0.9 (0.6, 1.1)	1.9 (0.5, 3.3)	0.5 (0.1, 0.9)	3.6 (2.1, 5.1)	1.0 (0.6, 1.5)	2.6 ^u^ (0.5, 4.8)	0.8 ^u^ (0.1, 1.4)	2.9 (1.8, 4.0)	1.4 (0.6, 2.1)	-	-	1.6 (0.6, 2.5)	1.3 (0.6, 2.1)
**Playing, Sports, Exercising**	820	10.3 (9.0, 11.5)	3.9 (3.4, 4.4)	30.7 (26.2, 35.2)	7.9 (6.5, 9.3)	13.2 (10.2, 16.2)	3.8 (2.9, 4.7)	8.3 (4.9, 11.7)	2.4 (1.4, 3.5)	3.5 (2.0, 5.0)	1.7 (1.0, 2.4)	2.7 (1.1, 4.4)	1.3 (0.4, 2.1)	1.5 (0.7, 2.3)	1.3 (0.6, 2.0)
**Other**	27	0.3 (0.1, 0.5)	0.1 (0.1, 0.2)	-	-	-	-	-	-	-	-	-	-	-	-
**Going Up/Down Stairs**	937	11.7 (10.5, 12.9)	4.4 (3.9, 4.9)	6.0 (4.0, 8.0)	1.6 (1.0, 2.1)	18.9 (15.1, 22.6)	5.4 (4.2, 6.6)	9.4 (6.3, 12.5)	2.8 (1.8, 3.7)	13.8 (11.2, 16.4)	6.5 (5.2, 7.9)	8.9 (5.2, 12.5)	4.1 (2.3, 5.9)	10.2 (8.1, 12.4)	8.8 (6.7, 10.9)
**Working From Elevation**	385	4.8 (4.0, 5.7)	1.8 (1.5, 2.2)	10.8 (7.4, 14.2)	2.8 (1.8, 3.7)	1.3 (0.6, 2.0)	0.4 (0.2, 0.6)	13.4 (9.8, 17.0)	3.9 (2.6, 5.2)	2.0 (1.0, 2.9)	0.9 (0.5, 1.4)	4.4 (1.9, 7.0)	2.0 (0.8, 3.3)	1.2 (0.6, 1.9)	1.1 (0.5, 1.7)
**Going Up/Down or Working From A Ladder**	258	3.2 (2.6, 3.9)	1.2 (0.9, 1.5)	4.9 (2.7, 7.2)	1.3 (0.7, 1.9)	0.8 ^u^ (0.2, 1.4)	0.2 ^u^ (0.1, 0.4)	11.2 (7.7, 14.6)	3.3 (2.0, 4.5)	1.4 (0.6, 2.3)	0.7 (0.3, 1.1)	3.7 ^u^ (1.3, 6.2)	1.7 ^u^ (0.6, 2.8)	1.1 (0.5, 1.7)	0.9 (0.4, 1.5)
**Working On Roof, Tree, or Elevated Surface**	113	1.4 (0.9, 1.9)	0.5 (0.3, 0.7)	5.4 (2.7, 8.1)	1.4 (0.7, 2.1)	-	-	2.0 ^u^ (0.6, 3.5)	0.6 ^u^ (0.2, 1.0)	-	-	-	-	-	-
**Other Activities**	442	5.5 (4.7, 6.3)	2.1 (1.8, 2.4)	3.7 (2.0, 5.3)	0.9 (0.5, 1.4)	4.3 (2.7, 5.8)	1.2 (0.8, 1.7)	5.2 (3.1, 7.2)	1.5 (0.8, 2.2)	5.6 (3.9, 7.2)	2.6 (1.8, 3.5)	11.1 (7.2, 14.9)	5.1 (3.1, 7.1)	6.2 (4.6, 7.8)	5.3 (3.9, 6.8)
**Getting On/Off or In/Out of Transportation Vehicle**	94	1.2 (0.8, 1.6)	0.4 (0.3, 0.6)	-	-	0.7 ^u^ (0.2, 1.2)	0.2 ^u^ (0.0, 0.4)	1.9 ^u^ (0.6, 3.3)	0.6 ^u^ (0.2, 1.0)	0.9 ^u^ (0.3, 1.6)	0.4 ^u^ (0.1, 0.8)	3.3 ^u^ (0.5, 6.1)	1.5 ^u^ (0.1, 2.9)	-	-
**Stepping Over**	66	0.8 (0.5, 1.1)	0.3 (0.2, 0.4)	-	-	0.7 ^u^ (0.1, 1.3)	0.2 ^u^ (0.0, 0.4)	-	-	1.0 ^u^ (0.3, 1.7)	0.5 ^u^ (0.1, 0.8)	1.9 ^u^ (0.5, 3.3)	0.9 ^u^ (0.2, 1.6)	0.9 (0.4, 1.4)	0.8 (0.3, 1.2)
**Bending**	42	0.5 (0.3, 0.8)	0.2 (0.1, 0.3)	-	-	-	-	-	-	0.5 ^u^ (0.1, 0.9)	0.2 ^u^ (0.0, 0.4)	-	-	1.1 (0.4, 1.8)	0.9 (0.3, 1.6)
**Sleeping**	86	1.1 (0.7, 1.4)	0.4 (0.3, 0.5)	-	-	-	-	-	-	0.6 ^u^(0.1, 1.1)	0.3 ^u^ (0.0, 0.5)	2.7 ^u^ (1.1, 4.4)	1.3 ^u^ (0.4, 2.1)	1.9 (0.9, 2.8)	1.6 (0.8, 2.5)
**Standing On Desk, Bed, Chair, Stool, Etc.**	74	0.9 (0.6, 1.3)	0.4 (0.2, 0.5)	-	-	1.4 ^u^ (0.4, 2.4)	0.4 ^u^ (0.1, 0.7)	-	-	1.4 (0.4, 2.4)	0.7 (0.2, 1.2)	-	-	0.9 (0.3, 1.4)	0.7 (0.3, 1.2)
**Reaching**	62	0.8 (0.5, 1.1)	0.3 (0.2, 0.4)	-	-	0.6 ^u^ (0.1, 1.0)	0.2 ^u^ (0.0, 0.3)	-	-	0.8 ^u^ (0.1, 1.5)	0.4 ^u^ (0.0, 0.7)	1.7 (0.3, 3.0)	0.8 (0.1, 1.4)	0.9 (0.3, 1.5)	0.8 (0.3, 1.3)
**Carrying/Loading/Lifting/Lowering/ Pushing/Pulling**	250	3.1 (2.5, 3.7)	1.2 (1.0, 1.4)	3.8 (2.3, 5.4)	1.0 (0.6, 1.4)	1.6 (0.9, 2.4)	0.5 (0.2, 0.7)	4.6 (2.3, 6.8)	1.3 (0.7, 2.0)	3.4 (2.1, 4.8)	1.6 (1.0, 2.3)	2.6 ^u^ (0.9, 4.4)	1.2 ^u^ (0.4, 2.0)	3.0 (1.8, 4.2)	2.6 (1.5, 3.7)
**Carrying**	162	2.0 (1.6, 2.5)	0.8 (0.6, 0.9)	2.3 (1.1, 3.6)	0.6 (0.3, 0.9)	1.4 (0.7, 2.0)	0.4 (0.2, 0.6)	3.2 (1.3, 5.1)	0.9 (0.4, 1.5)	2.1 (1.0, 3.2)	1.0 (0.5, 1.5)	-	-	1.9 (1.0, 2.8)	1.6 (0.9, 2.4)
**Loading/Lifting/Lowering/Pushing/Pulling**	89	1.1 (0.7, 1.5)	0.4 (0.3, 0.6)	1.5 (0.5, 2.5)	0.4 (0.1, 0.6)	-	-	1.4 (0.1, 2.6)	0.4 (0.0, 0.8)	1.3 (0.5, 2.1)	0.6 (0.2, 1.0)	-	-	1.1 (0.2, 2.0)	1.0 (0.2, 1.7)
**Activities Not Specified or Unknown**	1,405	17.6 (16.1, 19.0)	6.6 (6.0, 7.3)	15.0 (11.6, 18.4)	3.9 (2.9, 4.8)	17.2 (13.6, 20.7)	4.9 (3.7, 6.1)	17.5 (13.6, 21.4)	5.1 (3.8, 6.5)	16.3 (13.5, 19.2)	7.8 (6.2, 9.3)	20.0 (15.5, 24.6)	9.2 (6.7, 11.7)	20.3 (17.5, 23.1)	17.4 (14.6, 20.3)

^a^ in thousands, SA = weighted Sampled Adults per year, FI = weighted average fall-related injuries per year, IR = Incidence Rate Per 1000 person-years, CI = Confidence Interval

* Proportions may not add up to 100% as cells with unweighted count of 10 or less for the overall category and 5 or less for age-gender groups are not shown

^u^ unweighted count between 6–10, the estimates may be unstable

### Initiating event

**Fig 3:**
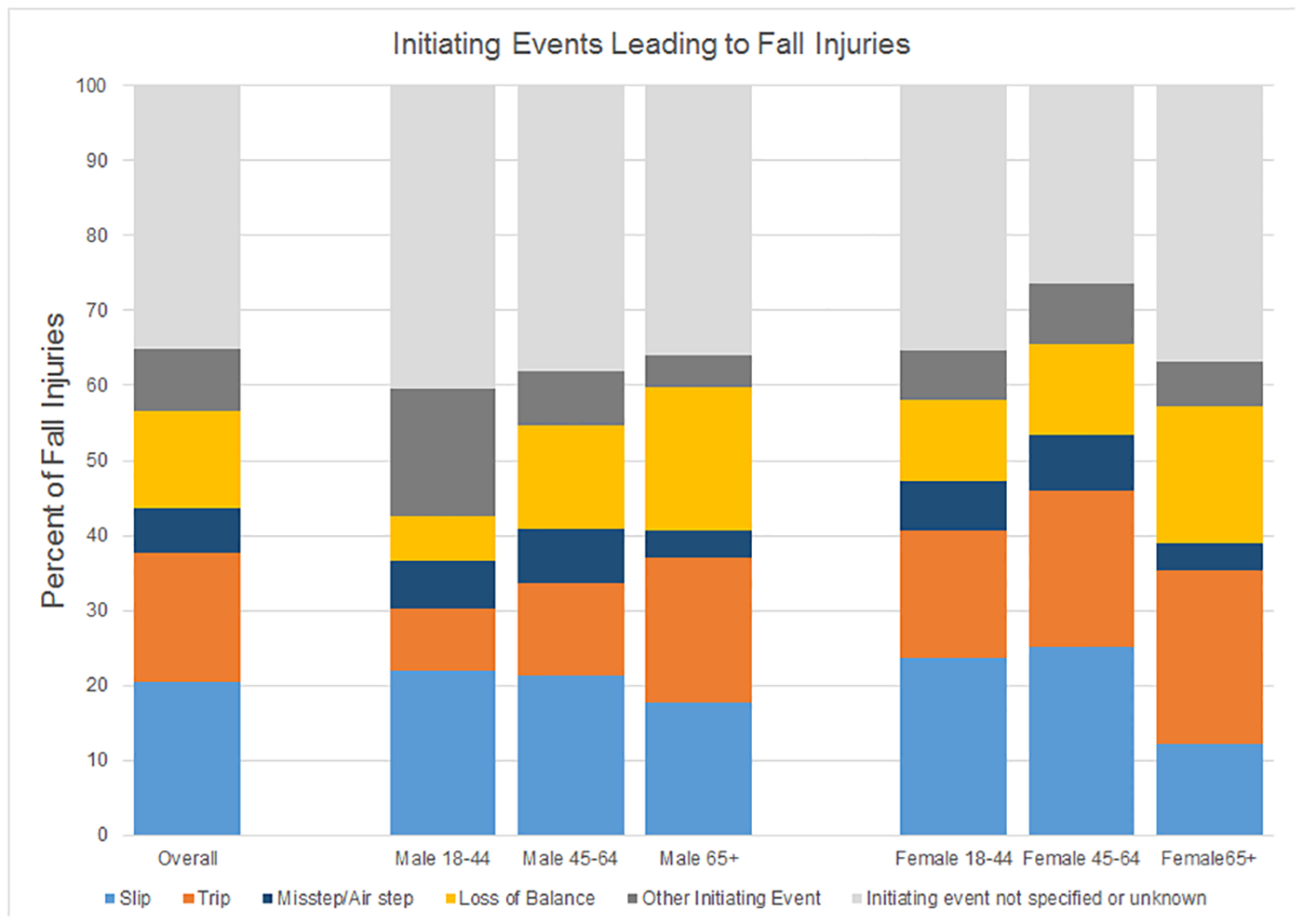
Initiating events leading to fall injuries (%).

Slips (IR = 7.7 per 1000 person-years, 95% CI 7.1, 8.3), trips (IR = 6.5 per 1000 person-years, 95% CI 5.9, 7.1) and loss of balance without slip, trip, or misstep (IR = 4.9 per 1000 person-years) were the three most common initiating events leading to FI (Table 4). For young and middle-aged adults, particularly men, a greater proportion of FI was due to slipping as compared to tripping, whereas for older adults more FI resulted from tripping (Fig 3). In each age group, females reported a higher proportion of FI from tripping as compared to men. The proportion of FI due to loss of balance (without slip, trip, or misstep) increased with age in both genders.

**Table 4:** Initiating events leading to fall-related injuries (proportion* (%) and incidence rate (IR) per 1000 person-years and their 95% confidence intervals) by age and gender.

INITIATING EVENT	Overall			18–44 years				45–64 years				65+ years			
				Males (SA = 54,348 FI = 1,396)^a^		Females (SA = 55,264 FI = 1,576)^a^		Males (SA = 33,104 FI = 972)^a^		Females (SA = 35,159 FI = 1,666)^a^		Males (SA = 14,788 FI = 679)^a^		Females (SA = 19,844 FI = 1,706)^a^	
	FI (000)	% (95% CI)	IR (95% CI)	% (95% CI)	IR (95% CI)	% (95% CI)	IR (95% CI)	% (95% CI)	IR (95% CI)	% (95% CI)	IR (95% CI)	% (95% CI)	IR (95% CI)	% (95% CI)	IR (95% CI)
**Slip**	1,632	20.4 (19.1, 21.8)	7.7 (7.1, 8.3)	21.9 (18.4, 25.5)	5.6 (4.5, 6.8)	23.6 (19.9, 27.3)	6.7 (5.5, 7.9)	21.4 (16.4, 26.3)	6.3 (4.6, 8.0)	25.1 (21.7, 28.4)	11.9 (9.9, 13.9)	17.8 (13.2, 22.5)	8.2 (5.8, 10.6)	12.1 (9.9, 14.4)	10.4 (8.3, 12.5)
**Trip**	1,381	17.3 (15.9, 18.6)	6.5 (5.9, 7.1)	8.3 (6.0, 10.5)	2.1 (1.5, 2.8)	17.1 (13.7, 20.5)	4.9 (3.8, 5.9)	12.3 (8.8, 15.7)	3.6 (2.5, 4.7)	20.9 (18.0, 23.8)	9.9 (8.3, 11.5)	19.3 (15.0, 23.7)	8.9 (6.5, 11.2)	23.3 (20.1, 26.4)	20.0 (16.7, 23.3)
**Misstep/Air step**	469	5.9 (5.1, 6.7)	2.2 (1.9, 2.5)	6.5 (4.4, 8.5)	1.7 (1.1, 2.2)	6.6 (4.4, 8.8)	1.9 (1.2, 2.5)	7.2 (4.0, 10.3)	2.1 (1.2, 3.1)	7.4 (5.2, 9.5)	3.5 (2.4, 4.5)	3.5 (1.0, 5.9)	1.6 (0.4, 2.7)	3.5 (2.3, 4.6)	3.0 (2.0, 4.0)
**Loss of Balance**	1,034	12.9 (11.7, 14.1)	4.9 (4.4, 5.4)	5.9 (3.9, 7.9)	1.5 (1.0, 2.0)	10.8 (7.8, 13.8)	3.1 (2.1, 4.1)	13.8 (9.6, 18.0)	4.1 (2.7, 5.4)	12.2 (9.7, 14.6)	5.8 (4.5, 7.0)	19.1 (14.6, 23.7)	8.8 (6.4, 11.2)	18.4 (15.8, 21.1)	15.9 (13.2, 18.5)
**Other Initiating Event**	205	2.6 (2.0, 3.2)	1.0 (0.7, 1.2)	3.1 (1.1, 5.2)	0.8 (0.3, 1.4)	2.0 (1.0, 2.9)	0.6 (0.3, 0.9)	2.0 (0.8, 3.3)	0.6 (0.2, 1.0)	3.9 (2.5, 5.3)	1.9 (1.0, 2.7)	-	-	2.4 (1.3, 3.6)	2.1 (1.1, 3.1)
**Break in Structure**	48	0.6 (0.4, 0.8)	0.2 (0.1, 0.3)	-	-	-	-	-	-	0.7^u^ (0.0, 1.5)	0.3 ^u^ (0.0, 0.7)	-	-	-	-
**Fall Through Surface**	38	0.5 (0.1, 0.8)	0.2 (0.0, 0.3)	-	-	-	-	-	-	-	-	-	-	-	-
**Missed The Seat**	16	0.2 (0.1, 0.3)	0.1 (0.0, 0.1)	-	-	-	-	-	-	0.5 (0.1, 0.9)	0.2 (0.0, 0.4)	-	-	-	-
**Violence or Animal/ Insect Involved**	74	0.9 (0.6, 1.2)	0.4 (0.2, 0.5)	-	-	0.9 ^u^ (0.2, 1.6)	0.3 ^u^ (0.1, 0.5)	-	-	1.6 (0.7, 2.4)	0.7 (0.3, 1.2)	-	-	1.2 ^u^ (0.3, 2.1)	1.0 ^u^ (0.2, 1.8)
**Animal/Insect**	63	0.8 (0.5, 1.1)	0.3 (0.2, 0.4)	-	-	0.8 ^u^ (0.1, 1.5)	0.2 ^u^ (0.0, 0.4)	-	-	1.3 (0.5, 2.0)	0.6 (0.2, 1.0)	-	-	1.2 ^u^ (0.3, 2.1)	1.0 ^u^ (0.2, 1.8)
**Supporting surface rolled, tipped, slid**	110	1.4 (1.0, 1.8)	0.5 (0.4, 0.7)	1.8 (0.6, 2.9)	0.5 (0.2, 0.7)	0.7 ^u^ (0.2, 1.3)	0.2 ^u^ (0.1, 0.4)	2.4 (0.8, 3.9)	0.7 (0.2, 1.2)	1.1 ^u^ (0.2, 2.1)	0.5 ^u^ (0.1, 1.0)	-	-	1.4 (0.8, 2.0)	1.2 (0.7, 1.8)
**Jumped**	94	1.2 (0.8, 1.6)	0.4 (0.3, 0.6)	3.8 (2.2, 5.4)	1.0 (0.6, 1.4)	1.3 (0.4, 2.1)	0.4 (0.1, 0.6)	-	-	-	-	-	-	-	-
**Contact with objects/equipment**	175	2.2 (1.6, 2.8)	0.8 (0.6, 1.1)	6.3 (3.7, 8.9)	1.6 (0.9, 2.3)	2.0 (1.0, 3.1)	0.6 (0.3, 0.9)	2.2 ^u^ (0.4, 4.0)	0.7 ^u^ (0.1, 1.2)	0.9 ^u^ (0.6, 1.3)	0.5 ^u^ (0.1, 0.8)	-	-	0.8 ^u^ (0.2, 1.3)	0.7 ^u^ (0.2, 1.1)
**Struck By Something/Someone**	102	1.3 (0.8, 1.7)	0.5 (0.3, 0.7)	4.4 (2.2, 6.6)	1.1 (0.5, 1.7)	0.6 ^u^ (0.1, 1.2)	0.2 ^u^ (0.0, 0.3)	1.6 ^u^ (0.0, 3.2)	0.5 ^u^ (0.0, 1.0)	-	-	-	-	0.6 ^u^ (0.1, 1.0)	0.5 ^u^ (0.1, 0.9)
**Struck Against Something**	67	0.8 (0.5, 1.2)	0.3 (0.2, 0.5)	1.6 ^u^ (0.3, 3.0)	0.4 ^u^ (0.1, 0.8)	1.3 ^u^ (0.3, 2.2)	0.4 ^u^ (0.1, 0.6)	-	-	0.7 ^u^ (0.5, 0.9)	0.3 ^u^ (0.0, 0.7)	-	-	-	-
**Contact Unknown**	6	0.1 (0.0, 0.2)	0.0 (0.0, 0.1)	-	-	-	-	-	-	-	-	-	-	-	-
**Overexertion**	85	1.1 (0.7, 1.4)	0.4 (0.3, 0.5)	1.8 (0.7, 2.9)	0.5 (0.2, 0.8)	-	-	-	-	1.1 ^u^ (0.3, 1.9)	0.5 ^u^ (0.1, 0.9)	1.8 ^u^ (0.3, 3.3)	0.8 ^u^ (0.1, 1.5)	0.9 (0.3, 1.4)	0.7 (0.3, 1.2)
**Initiating event not specified or unknown**	2,807	35.1 (33.4, 36.8)	13.2 (12.3, 14.1)	40.5 (35.8, 45.3)	10.4 (8.8, 12.0)	35.4 (31.7, 39.1)	10.1 (8.7, 11.5)	38.0 (32.3, 43.6)	11.1 (8.8, 13.5)	26.5 (22.9, 30.2)	12.6 (10.6, 14.6)	35.9 (30.2, 41.6)	16.5 (12.7, 20.3)	36.9 (33.3, 40.5)	31.7 (27.6, 35.8)

^**a**^ in thousands, SA = weighted Sampled Adults per year, FI = weighted average fall-related injuries per year, IR = Incidence Rate Per 1000 person-years, CI = Confidence Interval

* Proportions may not add up to 100% as cells with unweighted count of 10 or less for the overall category and 5 or less for age-gender groups are not shown

^u^ unweighted count between 6–10, the estimates may be unstable

### Hazards

Narrative analysis identified one or more hazards for 61% of the FI (Hazard Not Specified/Unknown = 39.1%, Table 5). Large objects (15.2%), stairs/steps (13.6%), surface contamination (13.1%), and elevation equipment/structure/machine/vehicle (10.9%) were the most common hazards. Among men, elevation equipment/structures/machine/vehicle were reported to be the more common fall hazards, as compared to women in the same age group. Older men and women reported, respectively, 17.6% and 17.3% of all fall-related injuries due to large objects as hazards. This was particularly due to fall-related injuries involving chair/bed/other furniture.

**Table 5:** Fall hazards for fall-related injuries (proportion (%) and incidence rate (IR) per 1000 person-years and their 95% confidence intervals) by age and gender.

HAZARD	Overall			18–44 years				45–64 years				65+ years			
				Males (SA = 54,348 FI = 1,396)^a^		Females (SA = 55,264 FI = 1,576)^a^		Males (SA = 33,104 FI = 972)^a^		Females (SA = 35,159 FI = 1,666)^a^		Males (SA = 14,788 FI = 679)^a^		Females (SA = 19,844 FI = 1,706)^a^	
	FI (000)	% (95% CI)	IR (95% CI)	% (95% CI)	IR (95% CI)	% (95% CI)	IR (95% CI)	% (95% CI)	IR (95% CI)	% (95% CI)	IR (95% CI)	% (95% CI)	IR (95% CI)	% (95% CI)	IR (95% CI)
**Surface Contamination**	1,050	13.1 (11.9, 14.3)	4.9 (4.4, 5.5)	13.5 (10.3, 16.8)	3.5 (2.6, 4.4)	15.2 (12.0, 18.3)	4.3 (3.4, 5.3)	14.0 (10.4, 17.7)	4.1 (2.9, 5.3)	18.4 (15.5, 21.4)	8.7 (7.0, 10.5)	9.6 (6.1, 13.2)	4.4 (2.7, 6.1)	6.6 (4.9, 8.3)	5.7 (4.2, 7.2)
**Liquid/Water**	482	6.0 (5.2, 6.9)	2.3 (1.9, 2.6)	4.4 (2.3, 6.6)	1.1 (0.6, 1.7)	7.1 (5.0, 9.1)	2.0 (1.4, 2.6)	4.7 (2.6, 6.7)	1.4 (0.8, 2.0)	10.7 (8.4, 13.0)	5.1 (3.8, 6.4)	4.2 (1.5, 6.9)	1.9 (0.7, 3.2)	3.3 (2.2, 4.4)	2.8 (1.9, 3.8)
**Grease/Oil**	45	0.6 (0.3, 0.9)	0.2 (0.1, 0.3)	-	-	1.1 ^u^ (0.4, 1.8)	0.3 ^u^ (0.1, 0.6)	-	-	-	-	-	-	-	-
**Ice/Snow**	494	6.2 (5.3, 7.1)	2.3 (2.0, 2.7)	8.1 (5.7, 10.5)	2.1 (1.4, 2.7)	6.7 (4.4, 9.0)	1.9 (1.2, 2.6)	7.9 (5.0, 10.9)	2.3 (1.4, 3.3)	6.8 (4.9, 8.7)	3.2 (2.2, 4.2)	5.4 (3.0, 7.8)	2.5 (1.4, 3.6)	2.9 (1.8, 3.9)	2.5 (1.5, 3.4)
**Stairs/Steps/Escalator/Elevator**	1,110	13.9 (12.6, 15.2)	5.2 (4.7, 5.8)	7.8 (5.4, 10.2)	2.0 (1.4, 2.7)	21.3 (17.4, 25.1)	6.1 (4.8, 7.3)	11.5 (8.2, 14.7)	3.4 (2.3, 4.4)	15.8 (13.1, 18.6)	7.5 (6.1, 9.0)	10.8 (7.0, 14.6)	5.0 (3.0, 6.9)	12.7 (10.3, 15.1)	10.9 (8.6, 13.2)
**Stairs/Steps**	1,090	13.6 (12.4, 14.9)	5.1 (4.6, 5.7)	7.6 (5.3, 10.0)	2.0 (1.3, 2.6)	21.0 (17.2, 24.8)	6.0 (4.8, 7.2)	11.5 (8.2, 14.7)	3.4 (2.3, 4.4)	15.7 (13.0, 18.4)	7.4 (6.0, 8.9)	10.8 (7.0, 14.6)	5.0 (3.0, 6.9)	12.1 (9.8, 14.4)	10.4 (8.1, 12.7)
**Elevation Equipment /Structure/Machine/Vehicle**	869	10.9 (9.6, 12.2)	4.1 (3.6, 4.6)	19.7 15.3, 24.0)	5.1 (3.8, 6.3)	5.8 (3.7, 7.9)	1.7 (1.0, 2.3)	20.2 (15.7, 24.8)	5.9 (4.3, 7.6)	7.7 (5.5, 9.8)	3.6 (2.6, 4.7)	13.5 (9.0, 18.1)	6.2 (3.9, 8.5)	5.1 (3.6, 6.7)	4.4 (3.0, 5.8)
**Ladder**	266	3.3 (2.7, 4.0)	1.3 (1.0, 1.5)	5.1 (2.8, 7.4)	1.3 (0.7, 1.9)	0.9 ^u^ (0.3, 1.5)	0.3 ^u^ (0.1, 0.4)	11.5 8.0, 15.0)	3.4 (2.1, 4.7)	1.4 (0.6, 2.3)	0.7 (0.3, 1.1)	4.0 (1.5, 6.4)	1.8 (0.7, 3.0)	1.1 (0.5, 1.7)	0.9 (0.4, 1.5)
**Raised Platform/Porch/Deck**	273	3.4 (2.7, 4.1)	1.3 (1.0, 1.6)	4.8 (2.6, 7.0)	1.2 (0.7, 1.8)	2.8 (1.5, 4.2)	0.8 (0.4, 1.2)	2.0 (0.7, 3.4)	0.6 (0.2, 1.0)	4.2 (2.6, 5.8)	2.0 (1.2, 2.8)	3.7 (1.5, 5.9)	1.7 (0.7, 2.8)	2.7 (1.6, 3.8)	2.3 (1.4, 3.3)
**Skylight/Roof/Trees**	121	1.5 (1.0, 2.0)	0.6 (0.4, 0.8)	5.0 (2.4, 7.6)	1.3 (0.6, 2.0)	-	-	3.1 (1.4, 4.7)	0.9 (0.3, 1.5)	-	-	-	-	-	-
**Equipment/Machinery/Vehicle**	194	2.4 (1.8, 3.1)	0.9 (0.7, 1.2)	4.3 (2.3, 6.3)	1.1 (0.6, 1.6)	1.6 ^u^ (0.1, 3.2)	0.5 ^u^ (0.0, 0.9)	3.2 (1.2, 5.3)	1.0 (0.3, 1.6)	1.7 (0.7, 2.8)	0.8 (0.3, 1.3)	4.2 (1.3, 7.0)	1.9 (0.5, 3.4)	1.2 ^u^ (0.3, 2.0)	1.0 ^u^ (0.3, 1.7)
**Uneven Ground or Surface**	406	5.1 (4.3, 5.9)	1.9 (1.6, 2.2)	4.5 (2.7, 6.3)	1.2 (0.7, 1.6)	5.5 (3.8, 7.2)	1.6 (1.1, 2.1)	5.3 (2.7, 7.8)	1.6 (0.8, 2.3)	6.9 (4.8, 8.9)	3.3 (2.1, 4.4)	3.5 (1.2, 5.8)	1.6 (0.6, 2.7)	3.9 (2.7, 5.2)	3.4 (2.2, 4.6)
**Cracked/Uneven Pavement or Sidewalk**	90	1.1 (0.7, 1.5)	0.4 (0.3, 0.6)	-	-	1.3 ^u^ (0.3, 2.4)	0.4 ^u^ (0.1, 0.7)	-	-	1.8 (0.6, 2.9)	0.8 (0.3, 1.4)	-	-	1.5 (0.7, 2.4)	1.3 (0.5, 2.1)
**Hole/Pot Hole**	178	2.2 (1.7, 2.7)	0.8 (0.6, 1.0)	2.4 (1.1, 3.7)	0.6 (0.3, 1.0)	3.0 (1.7, 4.2)	0.9 (0.5, 1.2)	2.9 0.8, 4.9)	0.8 (0.2, 1.4)	3.4 (2.0, 4.7)	1.6 (0.8, 2.4)	-	-	0.6 ^u^ (0.1, 1.1)	0.5 ^u^ (0.1, 0.9)
**Large Object**	1,212	15.2 (13.7, 16.6)	5.7 (5.1, 6.3)	12.7 (9.0, 16.5)	3.3 (2.2, 4.3)	11.1 (8.5, 13.7)	3.2 (2.4, 4.0)	16.2 (11.2, 21.3)	4.8 (3.1, 6.5)	17.2 (14.2, 20.3)	8.2 (6.5, 9.8)	17.6 (13.2, 22.0)	8.1 (5.7, 10.4)	17.3 (14.6, 20.1)	14.9 (12.3, 17.5)
**Construction Debris, Equipment and/or Material**	104	1.3 (0.8, 1.9)	0.5 (0.3, 0.7)	3.8 (1.6, 6.1)	1.0 (0.4, 1.6)	-	-	3.1 ^u^ (1.0, 5.2)	0.9 ^u^ (0.3, 1.6)	-	-	-	-	-	-
**Packages/Products**	133	1.7 (1.1, 2.3)	0.6 (0.4, 0.9)	1.5 ^u^ (0.6, 2.4)	0.4 ^u^ (0.1, 0.7)	1.4 (0.5, 2.2)	0.4 (0.2, 0.6)	3.1 ^u^ (-0.7, 6.9)	0.9 ^u^ (-0.2, 2.0)	1.7 (0.7, 2.7)	0.8 (0.4, 1.3)	-	-	1.6 (0.9, 2.4)	1.4 (0.7, 2.1)
**Chair/Bed/ Other Furniture**	498	6.2 (5.4, 7.1)	2.3 (2.0, 2.7)	2.3 (1.0, 3.6)	0.6 (0.3, 0.9)	4.8 (2.9, 6.7)	1.4 (0.8, 1.9)	5.8 (3.2, 8.3)	1.7 (0.9, 2.5)	6.4 (4.5, 8.2)	3.0 (2.1, 4.0)	8.5 (5.4, 11.7)	3.9 (2.3, 5.5)	10.0 (7.8, 12.2)	8.6 (6.6, 10.6)
**Bathtub/Shower, Toilet/Potty Chair**	207	2.6 (2.0, 3.2)	1.0 (0.7, 1.2)	1.5 ^u^ (0.3, 2.6)	0.4 ^u^ (0.1, 0.7)	2.0 (0.8, 3.2)	0.6 (0.2, 0.9)	1.1 ^u^ (0.2, 2.0)	0.3 ^u^ (0.1, 0.6)	4.0 (2.3, 5.7)	1.9 (1.1, 2.8)	4.0 (1.3, 6.7)	1.8 (0.6, 3.1)	3.0 (1.9, 4.1)	2.6 (1.7, 3.5)
**Cable/Wire/Rope/Hose**	105	1.3 (0.9, 1.7)	0.5 (0.3, 0.7)	1.2 ^u^ (0.0, 2.3)	0.3 ^u^ (0.0, 0.6)	-	-	-	-	2.2 (1.1, 3.3)	1.0 (0.5, 1.6)	-	-	1.4 (0.4, 2.4)	1.2 (0.4, 2.1)
**Small Objects**	383	4.8 (3.9, 5.7)	1.8 (1.5, 2.2)	3.2 (1.6, 4.9)	0.8 (0.4, 1.3)	3.7 (2.3, 5.1)	1.1 (0.6, 1.5)	6.7 (2.7, 10.8)	2.0 (0.7, 3.3)	5.1 (3.5, 6.8)	2.4 (1.6, 3.2)	4.6 (2.2, 7.1)	2.1 (1.0, 3.3)	5.7 (4.0, 7.5)	4.9 (3.3, 6.5)
**Clutter**	81	1.0 (0.7, 1.4)	0.4 (0.3, 0.5)	1.3 ^u^ (0.3, 2.3)	0.3 ^u^ (0.1, 0.6)	0.7 ^u^ (0.1, 1.2)	0.2 ^u^ (0.0, 0.4)	-	-	1.2 ^u^ (0.4, 2.0)	0.6 ^u^ (0.2, 1.0)	-	-	1.2 (0.4, 2.0)	1.1 (0.4, 1.8)
**Other**	175	2.2 (1.6, 2.7)	0.8 (0.6, 1.0)	-	-	1.7 (0.7, 2.7)	0.5 (0.2, 0.8)	2.1 ^u^ (0.6, 3.6)	0.6 ^u^ (0.2, 1.1)	1.8 (0.8, 2.8)	0.9 (0.4, 1.3)	-	-	3.4 (1.9, 4.8)	2.9 (1.6, 4.2)
**Curbs/Car Stops**	99	1.2 (0.9, 1.6)	0.5 (0.3, 0.6)	1.3 ^u^ (0.4, 2.2)	0.3 ^u^ (0.1, 0.6)	1.8 (0.8, 2.7)	0.5 (0.2, 0.8)	-	-	0.8 ^u^ (0.2, 1.5)	0.4 ^u^ (0.1, 0.7)	1.8 ^u^ (0.0, 3.7)	0.8 ^u^ (0.0, 1.7)	1.4 (0.5, 2.2)	1.2 (0.5, 1.9)
**Rug/Mat/Carpet Runner**	116	1.4 (1.0, 1.9)	0.6 (0.4, 0.7)	1.2 ^u^ (0.2, 2.1)	0.3 ^u^ (0.0, 0.6)	0.8 ^u^ (0.1, 1.4)	0.2 ^u^ (0.0, 0.4)	-	-	1.0 (0.5, 1.6)	0.5 (0.2, 0.8)	1.8 ^u^ (0.1, 3.6)	0.8 ^u^ (0.0, 1.7)	2.9 (1.7, 4.1)	2.5 (1.5, 3.6)
**Small Children/Animal/Pets/Insects**	281	3.5 (2.9, 4.1)	1.3 (1.1, 1.6)	2.0 (0.9, 3.1)	0.5 (0.2, 0.8)	5.3 (3.5, 7.1)	1.5 (1.0, 2.1)	1.5 ^u^ (0.3, 2.6)	0.4 ^u^ (0.1, 0.8)	5.2 (3.7, 6.7)	2.5 (1.7, 3.2)	-	-	3.5 (2.2, 4.8)	3.0 (1.8, 4.1)
**Small Children**	83	1.0 (0.7, 1.4)	0.4 (0.3, 0.5)	0.9 ^u^ (0.2, 1.7)	0.2 ^u^ (0.0, 0.4)	1.5 (0.5, 2.4)	0.4 (0.1, 0.7)	-	-	1.4 (0.6, 2.1)	0.6 (0.2, 1.1)	-	-	1.0 (0.4, 1.6)	0.8 (0.3, 1.4)
**Animal/Pets/Insects**	197	2.5 (1.9, 3.0)	0.9 (0.7, 1.1)	1.1 ^u^ (0.3, 1.9)	0.3 ^u^ (0.1, 0.5)	3.8 (2.3, 5.4)	1.1 (0.6, 1.6)	-	-	3.8 (2.5, 5.2)	1.8 (1.2, 2.5)	-	-	2.5 (1.3, 3.6)	2.1 (1.1, 3.1)
**Footwear**	131	1.6 (1.2, 2.1)	0.6 (0.4, 0.8)	1.0 ^u^ (0.1, 1.8)	0.3 ^u^ (0.0, 0.5)	2.8 (1.3, 4.3)	0.8 (0.3, 1.3)	-	-	2.1 (0.8, 3.4)	1.0 (0.4, 1.6)	-	-	1.7 (0.9, 2.4)	1.4 (0.8, 2.1)
**Other Hazard**	107	1.3 (0.9, 1.7)	0.5 (0.4, 0.7)	1.5 (0.3, 2.6)	0.4 (0.1, 0.7)	1.3 (0.5, 2.1)	0.4 (0.1, 0.6)	1.4 (0.3, 2.5)	0.4 (0.1, 0.7)	0.7 (0.1, 1.3)	0.3 (0.0, 0.6)	-	-	2.0 (0.9, 3.2)	1.7 (0.7, 2.8)
**Hazard Not Specified/ Unknown**	3,127	39.1 (37.3, 41.0)	14.7 (13.8, 15.7)	42.9 (38.2, 47.6)	11.0 (9.4, 12.7)	36.6 (32.3, 40.8)	10.4 (8.8, 12.1)	33.5 (27.9, 39.1)	9.8 (7.8, 11.9)	33.5 (29.9, 37.2)	15.9 (13.8, 18.0)	43.9 (37.4, 50.4)	20.2 (15.8, 24.5)	45.1 (41.5, 48.7)	38.8 (34.3, 43.3)

^a^ in thousands, SA = weighted Sampled Adults per year, FI = weighted average fall-related injuries per year, IR = Incidence Rate Per 1000 person-years, CI = Confidence Interval

^u^ unweighted count between 6–10, the estimates may be unstable

### Same vs. lower level, work-relatedness

About 20% of FI were due to falls to a lower level (1.6 million annually). This proportion ranged from 25% among middle-aged men (Table 6) to 14% in older women.

**Table 6:** Level and work-relatedness of fall-related injuries (proportion (%) and incidence rate (IR) per 1000 person-years and their 95% confidence intervals) by age and gender.

	Overall			18–44 years				45–64 years				65+ years			
				Males(SA = 54,348 FI = 1,396)^a^		Females(SA = 55,264 FI = 1,576)^a^		Males(SA = 33,104 FI = 972)^a^		Females(SA = 35,159 FI = 1,666)^a^		Males(SA = 14,788 FI = 679)^a^		Females(SA = 19,844 FI = 1,706)^a^	
	FI (000)	% (95% CI)	IR (95% CI)	% (95% CI)	IR (95% CI)	% (95% CI)	IR (95% CI)	% (95% CI)	IR (95% CI)	% (95% CI)	IR (95% CI)	% (95% CI)	IR(95% CI)	% (95% CI)	IR (95% CI)
**LEVEL**															
**Same Level**	6,425	80.4 (78.8, 81.9)	30.2 (28.8, 31.7)	75.6 (71.2, 80.0)	19.4 (17.2, 21.6)	78.6 (75.1, 82.1)	22.4 (20.1, 24.7)	75.2 (70.6, 79.8)	22.1 (18.8, 25.4)	82.6 (79.7, 85.6)	39.2 (35.8, 42.6)	81.9 (77.2, 86.5)	37.6 (31.8, 43.4)	86.0 (83.6, 88.3)	73.9 (67.7, 80.1)
**To Lower Level**	1,569	19.6 (18.1, 21.1)	7.4 (6.7, 8.0)	24.3 (19.9, 28.7)	6.2 (4.9, 7.6)	21.4 (17.9, 24.8)	6.1 (5.0, 7.2)	24.9 (20.4, 29.4)	7.3 (5.7, 9.0)	17.3 (14.3, 20.2)	8.2 (6.6, 9.8)	18.2 (13.7, 22.7)	8.4 (5.9, 10.8)	14.0 (11.7, 16.4)	12.1 (9.7, 14.4)
**WORK-RELATEDNESS**^b^															
**Working At Paid Job At Time Of Injury**	1,068	13.4 (12.0, 14.7)	5.0 (4.5, 5.6)	28.5 (24.0, 33.1)	7.3 (5.9, 8.8)	15.2 (12.0, 18.3)	4.3 (3.4, 5.3)	22.8 (17.5, 28.1)	6.7 (4.9, 8.5)	10.5 (8.2, 12.8)	5.0 (3.8, 6.2)	1.6.(0.1, 3.0)	0.7(0.0, 1.4)	1.4.(0.6, 2.2)	1.2.(0.5, 1.9)
**Not Working At Paid Job At Time Of Injury**	6,917	86.5 (85.2, 87.9)	32.6 (31.1, 34.1)	71.4 (66.8, 75.9)	18.3 (16.3, 20.4)	84.7 (81.6, 87.9)	24.2 (21.8, 26.6)	77.0 (71.7, 82.3)	22.6 (19.4, 25.8)	89.3 (87.0, 91.6)	42.3 (38.8, 45.8)	98.3 (96.8, 99.8)	45.2 (38.7, 51.6)	98.6.(97.8, 99.4)	84.8.(78.2, 91.4)
**WORK-RELATEDNESS**^c^															
**Working At Paid Job At Time Of Injury**	988	26.6 (25.5, 27.5)	7.2 (6.4, 8.1)	33.1 (31.1, 34.5)	8.0 (6.4, 9.6)	22.8 (20.4, 24.6)	5.9 (4.5, 7.3)	34.5 (32.3, 35.7)	8.2 (5.8, 10.5)	19.2 (16.9, 21)	7.2 (5.4, 8.9)	-	-	19.4 (11.4, 23.6)	10.2 (3.8, 16.7)
**Not Working At Paid Job At Time Of Injury**	2,726	73.4 (74.5, 72.5)	20 (18.7, 21.3)	66.9 (68.9, 65.5)	16.2 (14.1, 18.2)	77.2 (79.6, 75.4)	20 (17.7, 22.3)	65.5 (67.7, 64.3)	15.6 (12.2, 19)	80.8 (83.1, 79)	30.1 (26.6, 33.6)	88.4 (102.8, 83.3)	23.3 (14.2, 32.4)	80.6 (88.6, 76.4)	41.8 (29.7, 54)

^a^ in thousands

^b^ For the percent or incidence rate of fall-related injuries while working at a paid job the denominator included both workers and non-workers (to be consistent with other tables). These estimates would be higher if the denominator is limited only to workers.

^C^ = Sample restricted those who worked in the previous week or were employed

SA = weighted Sampled Adults per year, FALLRI = weighted average fall-related injuries per year, IR = Incidence Rate Per 1000 people

Of all FI, 13.4% occurred while working at a paid job (1.1 million FI annually). A higher proportion of FI were work-related among young and middle-aged men (28.5% and 22.8%, respectively, Table 6) compared to other age-gender groups. When the sample was restricted to those worked in the past week and/or were employed, 27% of all FI occurred while working and 73% of FI were not work-related (Table 6).

## Discussion

This study is the one of the first comprehensive studies to use a large, nationally representative dataset to examine circumstances of FI in community-dwelling adults in the U.S. We presented national estimates of annual FI associated with various circumstances among community-dwelling U.S adults. We also described circumstances of FI not only among older adults, but also young and middle-aged adults. We observed many differences in the circumstances of FI by age and gender, as well as circumstances that are pervasive regardless of gender and age.

### Place

We observed a higher proportion of outdoor FI as compared to indoor FI among young men, young women, and middle-aged men. For middle-aged women and older men, outdoor and indoor FI proportions were similar, and more indoor FI occurred among older women. Our findings are similar to observations from the Baltimore Longitudinal Study on Aging that young participants fell more outdoors, whereas the percentage of falls indoors increased from middle-age to older age.[15] Another recent study reported more outdoor falls among a sample of undergraduate students.[22] Likewise, Kelsey et al. found that among older adults, increasing age and female gender were associated with a higher number of indoor falls.[18]

Our findings of equal proportions of indoor and outdoor fall-related injuries among older men and more indoor fall-related injuries among older women are in contrast to the findings from studies of community-dwelling older adults in Canada, U.K., and Japan. [33–35] For example, 81% of injurious falls among older men and 51% of injurious falls among older women occurred outdoors in Japan. In previous studies more indoor falls were observed among older adults who were frail, had poor health status and were leading inactive lifestyle, whereas outdoor falls occurred more among active and mobile older adults. [18, 34]

Several studies have examined the circumstances of falls inside the home. [36, 37]The U.S. Centers for Disease Control and Prevention provides a checklist for in home fall prevention. [38] We also observed a substantial number and proportion of outdoor FI, and few studies have examined circumstances of outdoor falls.[14] Many outdoor falls occurred around the home, particularly among older men for whom 31% of FI occurred outdoors around the home. Keall et al. found that low-cost home modifications and repairs can reduce FI in the general population.[39] In their study, home modifications addressed hazards both inside and outside the home, and they consisted of: handrails for outside steps and internal stairs; other minor repairs to outside steps; repairs to window catches; grab rails for bathrooms and toilets; adequate outside lighting; high-visibility and slip-resistant edging for outside steps; fixing of lifted edges of carpets and mats; non-slip bathmats; and slip-resistant surfacing for outside surfaces such as decks.

About 10% of all FI occurred on outdoor paved surfaces (Fig 1). Snow and ice, unevenness of the surfaces, and lighting conditions could be some of the reasons for falls on these surfaces. [40] [14] [41] Thoughtful design, proper selection of materials and regular maintenance to address these hazards could help prevent falls on paved surfaces. We also observed that 26% of FI among young men occurred at “Outdoor park/recreation area/sports facility.” This finding is consistent with previous observations that, among a sample of undergraduate students, falls during athletic activities (including running, jogging, and sports) accounted for 21% of their falls.[22]

### Activity

Consistent with previous studies,[15, 16] walking was the predominant activity preceding FI in all age-gender groups (37% of all FI), except young men. In this study, walking was coded as default for any undefined activity when the narrative described a slip or trip with no additional information, and the high prevalence of walking may be attributed to the coding approach. However, other studies have also indicated that one-third to one-half of falls among people aged ≥65 years occur while walking, which is consistent with our observations that 40.5% and 41.5% of FI among older men and women, respectively, occurred while walking.[17, 42–45]

Walking is a leading leisure-time physical activity in the U.S., and in 2010, 62% of U.S. adults reported engaging in at least one bout of 10 minutes or more of transportation walking or leisure-time walking during the past seven days.[46] Physical activity is associated with several positive health outcomes, and there is increased momentum in public health and community planning to develop walkable neighborhoods and increase walking opportunities in neighborhoods.[47] However, increased exposure to walking may also increase the risk of falls and FI, and walking-only intervention studies have not been found to be effective in reducing the risk of falls among older adults.[48] Therefore, the campaign to encourage walking and to develop walkable neighborhoods should take safe infrastructure/fall protective design into consideration for people of all ages.

An estimated 937 thousand FI occurred annually while using the stairs. We found a higher proportion of FI while using the stairs for women as compared to men, and the proportion was the highest among young women (19%) followed by middle-aged (14%) and older (10%) women. Other studies have also found women to be at a higher risk of stair-related FI.[49– 50] Variation in step geometry, stair steepness, absence of hand rails, and short flights have been shown to be associated with increased risk of falls. [51–54] More research is needed on safe stair design, gender differences in stair negotiation [55–57], and how these factors may be accounted for in stair design with affordance for all users.[58]

We found 258 thousand annual FI related to ladder use, with 11.2% of FI among middle-aged men attributable to ladder use. D'Souza et al. reported 136,118 ladder-related injury cases treated annually in emergency departments.[59] Further, 11.2% of all fall-related injuries among middle-aged men (Incidence Rate 3.3 per 1000 person-years, 95% CI 2.0, 4.5) were attributable to ladder use. One study reported that 18% of ladder-FI required hospital admission.[60] The median length of hospital stay was one week, and the median duration of disability and unemployment was six weeks. [61] Lombardi et al. reported that ladder movement was the mechanism in 40% of falls in occupational settings.[62] The U.S. Occupation Safety and Health Administration provides guidance to safely use portable ladders. [63] Ladder falls lead to severe injuries, and more effort is needed to understand the mechanisms of ladder falls and potential prevention approaches.

### Initiating event

While more FI occurred from slipping in young and middle-aged adults, more FI occurred due to tripping in the older adults, which was consistent in both genders. Older adults report tripping as the primary initiating event leading to falls.[64] Laboratory research has also demonstrated increased foot clearance variability and increased probability of tripping among older adults. [65–66] Occupational studies, which primarily include young and middle-aged adults, report slipping as the primary cause of falls.[67] Verma et al. reported that increasing age was associated with increased injury risk from tripping-initiated falls among women workers over 45 years of age.[68]

Women were more likely to trip than men in any age group, which may be related to gender differences in gait.[69–71] However, it is unclear whether and how these gait differences make women more prone to tripping. Differences in footwear by gender may also contribute to increased risk of tripping among women.[72] The differences in the initiating events by age and gender suggest that fall prevention interventions (both at the individual level and at the environmental level) developed for one group may not be equally effective for everyone, and that more holistic and inclusive approaches will be necessary to prevent falls among all adults.

### Hazards

We identified external hazards for 61% of FI. Even among older adults, external hazards were identified for 55% of FI. These observations emphasize the role of extrinsic factors in the causation of falls and FI and the opportunity for developing interventions to prevent falls and injuries resulting from them.

Large objects, stairs and steps, and surface contamination were the three most common hazards noted for FI, respectively. Chair/bed/other furniture were the most common large-object hazards. The role of large objects in the precipitation of FI has not been well examined. Stairs and steps were the second most common hazard, and were discussed in the *Activity* section. Surface contamination (almost equally divided between Liquid/Water and Ice/Snow) were the third leading hazard. A number of occupational studies have identified surface contamination as a risk for slips and falls among workers.[73–74] However, very few studies have examined the role of surface contamination on the risk of falls in the general population.[15] As discussed in the *Place* section, a home hazard checklist from CDC and the components of home modification intervention from the study by Keall et al. provide guidelines to minimize hazard in and around the house. In the current study, the estimated incidence rate of fall-related injury due to ice and snow was 2.3 per 1000 persons per year in the U.S. A study conducted in Sweden reported 3.5 injuries per 1000 inhabitants per year due to slipping on ice or snow.[75] However, exposure to ice and snow may also be different in the two countries. Other than effective and timely removal of ice and snow, a few epidemiologic studies have reported that the use of anti-slip devices may reduce the risk of falls in the winter-time. [76–77]

### Work-relatedness and level of falls

About one million fall-related injuries occurred while working at a paid job. This estimate is higher than the U.S. Bureau of Labor Statistics report of 316,650 occupational nonfatal slips, trips, and fall-related injuries involving at least one day away from work in 2014 (http://www.bls.gov/news.release/osh2.nr0.htm). The difference in injury definition and the severity of injuries may be the primary reason for this difference. [25]

When the denominator was limited to workers, 27% of fall injuries were work-related and 73% of fall injuries were not work-related. Smith et al. argued that prevention of injury both on and off the job may be an effective way to improve worker health and safety. [78] Some injury circumstances may be unique to work, but for the majority of injuries the lessons learnt from one setting can be applied to another. [78–80]

When interpreting the percent or incidence rate of fall-related injuries while working at a paid job, it is important to remember that denominator in the current study included both workers and non-workers (to be consistent with other tables). These estimates would be higher if the denominator is limited only to workers.

Of all fall-related injuries, about 80% resulted from same-level falls (6.4 million FI annually) and 20% resulted in falls to a lower level (1.6 million FI annually). Among fall-related injuries that occurred while working a paid job, 25% were due to falls to lower level (data not shown). This proportion was 19% for fall-related injuries that occurred while not working a paid job.

### Strengths and limitations

The primary strength of this study is that it uses 14 years of data from a large-scale, nationally representative, population-based survey conducted through in-person interviews—the NHIS. The coding taxonomy developed for this study can be applied and further developed in future studies.

A primary limitation of narrative text analysis using surveillance data is that the data may be limited by the completeness and consistency of the available text. Words can be forgotten, lost, truncated, or abbreviated by those reporting and/or recording the narratives.[30] Any systematic difference in the reporting of circumstances could affect the estimation of the proportions and the incidence rates of FI related to those circumstances. Circumstances for a number of injury narratives could not be coded due to lack of information. It is not clear whether these narratives pertain to unique circumstances, or if the information was missing at random.

Secondly, we reported proportion of FI. The proportions sum to 100%, and any change in the proportion in one category will affect the proportions in other categories. The incidence rates do not have this limitation. However, the incidence rates presented in the study use the estimated within-group adult population for denominator (IR per 1000 population) and do not account for the participants’ actual time of exposure to particular circumstances. A higher incidence rate associated with a particular circumstance could be due to higher vulnerability, higher exposure, or both.

## Conclusions

This study detailed the circumstances of FI among community-dwelling U.S. adults and showed that there are important differences in not only the overall incidence rate of FI but also in the circumstances of FI by age and gender. While falls in older adults have been studied often, young and middle-aged adults also represent significant risk groups for FI. More emphasis is needed on exploring and designing interventions to mitigate FI in all adults. Finally, addressing both intrinsic factors *and* extrinsic circumstances is likely to significantly reduce morbidity related to FI in the U.S.

## Supporting information

S1 Table Appendix: Coding taxonomy. (DOCX)
