# Supplementary material for: Circumstances of fall-related injuries by age and gender among community-dwelling adults in the United States
Source: PLoS One. 2017 May 4;12(5):e0176561. doi: 10.1371/journal.pone.0176561 (PMC5417511; doi:10.1371/journal.pone.0176561)
Supplement: S1 Table — (DOCX) [file pone.0176561.s001.docx]

**S1 Appendix: Coding Taxonomy**

Instructions:

| 1. Narrative information supersedes the coded information, i.e. precoded variables in NHIS for place and activity |
| --- |
| 2. If nothing can be inferred from the verbatim text, the coder should try to infer appropriate information from the precoded variables in NHIS for place and activity |
| 3. Generally, a more specific code takes precedence over a less specific code. For example, if the narrative describes a person who fell while walking and ascending stairs, use "2.6 Going up/down stairs". |

**PLACE**

| **Place (1)** | **Description** |
| --- | --- |
| **INDOOR** | Mention of floor or indoor activities |
| 1.1.1 Public Restroom or bathroom | Restroom or bathroom that is not in a home |
| Home | Areas inside the home |
| 1.1.2.1 Bedroom | Bedroom |
| 1.1.2.2 Kitchen | Kitchen |
| 1.1.2.3 Restroom or bathroom | Restroom, bathroom |
| 1.1.2.9 Other/unknown parts of home (indoor) | Other parts of home (indoor) (default) |
| 1.1.5 Indoor sporting facility (ex: indoor ice arena) | Indoor sporting facility (ex: indoor ice arena) |
| 1.1.9 Other Indoor | Indoors other than home e.g. restaurant, shopping mall, health facility, etc. (indoor or preposition indicating indoor has to be specified in the text). Includes fall from chairs/stools if no further information is available. |
| **OUTDOOR** | Mention of ground, grass, dirt, pothole, etc. or outdoor activities |
| 1.2.1 Parking lot | Parking lot or garage |
| 1.2.2 Sidewalk | Sidewalk |
| 1.2.3 Curb | Curb of the street |
| 1.2.4 Street | Street |
| 1.2.5 Outdoor park/recreation area/sporting facility (ex:  Ski resort, football stadium) | Parks, lakes, or other recreation area including sports facility. By default, if indoor/gymnasium is not mentioned for sporting facilities, it will be considered as outdoor. |
| 1.2.6 Outdoor home (around house/yard) | Outdoor (home) such as around house/yard, driveways, etc. |
| 1.2.9 Other Outdoor | Other outdoors (not home) (outdoor or preposition indicating outdoor has to be specified in the text) |
| 1.98 Other/ Unknown (default) | Other/Unknown about outdoor or indoor place |

**ACTIVITY**

| **Activity (2)** | **Description** |
| --- | --- |
| 2.1 Moving from sitting to standing position | e.g. standing up from a chair, stool, couch, floor, bed or getting out of bed, etc. |
| 2.2 Moving from standing to sitting position | e.g. sitting down on a chair, stool, couch, floor, toilet, bed, etc. |
| 2.3 Standing | Standing on a ground/floor or in a tub/shower or in a bathroom (or without mention of any supporting structure to stand) but not transitioning |
| 2.4 Walking (speed not specified) (default) | Walking (speed not mentioned). Use this code for slips and trips where no other details are mentioned |
| Vigorous activity (includes Walking fast/Rushing/Running/Playing/Exercising) | Vigorous activity includes walking fast, running, rushing, hurrying, playing, etc. |
| 2.5.1 Walking fast/Rushing/Running | Walking fast in a hurry, rushing, or running |
| 2.5.2 Playing/Sporting/Exercising | Playing sports, swimming, skiing, snowboarding, working out, wrestling, horse playing, playing tug-of-war, dancing, martial arts, etc. If nothing can be inferred from the narrative refer to InjActivity1 and look for sports & exercise to code it as this. |
| 2.5.9 Unknown/Other (default) | Any other/unknown vigorous activity |
| 2.6 Going up/down stairs | Going up or down or working on the stairs with nothing in the hands or no mention of carrying (2.21.1) or lifting (2.21.3) |
| Using a ladder | Any activity while on a ladder or getting on or off a ladder with nothing in the hands |
| 2.7.2 Ladder-All ladder activities | Default for going up or down or working from ladder without mention of reaching (2.18) and without carrying (2.21.1) or lifting (2.21.3) |
| 2.8 Getting on/off or working from scaffolding | Going up or down or working from scaffolding |
| 2.9 Getting on/off or working from aerial lift equipment (e.g. boom lift, scissor lift, fork lift etc.) | Getting on/off or working from aerial lift equipment (e.g. boom lift, scissor lift, fork lift etc.) |
| 2.10 Getting on/off or in/out of transportation vehicle | Getting on/off or in/out of a transportation vehicle |
| 2.11 Stepping over | Stepping over something or over a child, pet. It requires significant lifting of leg. (for example: stepping into a bathtub.) |
| 2.12 Bending | Bending sideways, forward, or backward |
| 2.13 Sitting | Sitting on something but not transitioning (e.g. using toilet, wheelchair) |
| 2.14 Sleeping (includes sleep walking) | Sleeping (includes sleep walking), rolled out of bed while sleeping |
| 2.16 Standing up on a desk, bed, chair, stool, etc. | Standing up on a chair, desk bed, stool, etc. (e.g. to reach something up) presumed less than 6 feet. The support to stand up has to be mentioned specifically in the text. |
| 2.17 Working on a roof, tree, or other elevated surface | Working on a roof or elevated level (excludes, ladder, scaffolding, aerial lift equipment) presumed over 6 feet |
| 2.18 Reaching | Reaching to something without mention of any supporting structure |
| 2.19 Machine/equipment etc. (not transportation vehicle) | Working on a machine or other equipment that is not the transportation vehicle or are not moving on their wheels while the respondent was working or the motion of the machine or equipment is not specified |
| 2.20 Catching | Catching something or someone falling that overbalances the respondent |
| Carrying, loading, lifting, lowering, pushing, pulling |  |
| 2.21.1 Carrying | Carrying someone or something (above the ground/floor/steps) at the time of fall |
| 2.21.2 Loading | Loading something at the time fall |
| 2.21.3 Lifting | Lifting someone/something at the time of fall |
| 2.21.4 Lowering | Lowering someone/something at the time of fall |
| 2.21.5 Pushing | Pushing to move someone/something from one point to another |
| 2.21.6 Pulling | Pulling to move someone/something from one point to another |
| 2.98 Other/Unknown (default) | Other or Unknown activities not categorized into any category above. |
|  | |
|  | |

**INITIATING EVENT**

| **Initiating Event (3)** | **Description** |
| --- | --- |
| 3.1 Slip | Slip is specifically mentioned in the text. Fall on ice, or in snow, rain, or other slippery substances. "Fell in the bathroom/bathtub while bathing" shows some evidence of water on the surface and is coded as "3.1 Slip". "Fell in the bathroom/bathtub" is coded as "3.98 Other/Unknown (default)". |
| 3.2 Trip | Trip is specifically mentioned in the text. It could be due to stumble, falling over something or someone, stubbed toe, foot got caught, tripping over oneself, etc. |
| 3.3 Misstep/air-step | Misstep/air-step. e.g. fell off the porch, etc. (expecting a supporting surface that is not present, e.g. falling into a hole) |
| 3.4 Loss of balance (without slip, trip, or misstep) | Loss of balance. It may also be due to ankle/leg instability, twisted/rolled ankle, leg/knee gave away, or other medical conditions such as stroke. For elderly people who need assistive devices and they are walking without it, and no other information is given then the fall is also due to loss of balance (without slip, trip or misstep). "Fall while getting into a bathtub" which requires stepping over causes a person to balance on one leg and therefore fall related to this will be coded as "3.4 Loss of balance" unless water in mentioned ("3.1 Slip") |
| Break in structure or through surface |  |
| 3.5.1 Break in structure | Break in structure (chair, bed, or other furniture, shoe heels, running board, etc.) precedes fall. |
| 3.5.2 Fall through surface | Fall through surfaces on an otherwise intact structure or machine. It includes fall through existing surfaces such as skylights and roofs, and surfaces whose integrity fails due to rotten or weak flooring or ice or snow buildup. |
| 3.6 Slip and Trip | Slip and then trip or trip and then slip |
| 3.7 Supporting surface rolled, tipped, slid | Supporting surface could be anything that supports while standing (chair, stool, or other furniture that are not heavy and can be easily rolled, tipped, or slid), working (ladder, scaffold, etc.). |
| 3.8 Jumped | Jumping |
| 3.9 Missed the seat | Missed the seat while trying to sit on chair, stool, commode, etc. |
| Violence or animal/insect involved |  |
| 3.10.1 Violence | Fall due to act of violence involving someone. There has to be clear intent of purposefully inflicting violence to others or oneself. (e.g. Hitting, kicking, beating, biting, shoving, strangulation, gun shots, etc.) |
| 3.10.2 Animal/insect involved | Fall due to the involvement of animals or insects |
| 3.11 Fire/Explosion/harmful environment | Includes fire/explosion, electricity, high temperature, and other harmful environments that causes people to fall or pass out |
| Contact with objects/equipment | It has to precipitate fall and there has to be no mention of any slip or trip |
| 3.12.1 Struck by something/someone | There has to be contact with something that caused the fall. Contact with a person need not be a deliberate action by that person (e.g. struck by a ball while playing, pushed by someone as in a bus (when intent of the person is not specifically mentioned)) |
| 3.12.2 Struck against something | Struck against something (e.g. hit on a rock and fell) |
| 3.12.98 Contact (unknown) | All other contacts whose detail is unknown |
| 3.13 Overexertion | Lifting something too heavy and fell without any mention of loss of balance. |
| 3.98 Other/Unknown (default) | Other or unknown about the initiating events. Sports/playing without mention of slips and trips (e.g. "Fell while snowboarding" or "Fell while skiing"). However, if they "fell over a snowboard" then it should be coded as "3.2 Trip". "Sprained ankle" or other nature of injury gives no event information by itself. Code it as "3.98 Other/Unknown (default)". |
|  | |
|  | |

**HAZARDS**

| Select all that apply. Separate by a comma and a space  Code point of origin of slip/trip/falls as hazards and not the source of injury. For example, "Fell down the stairs and hit head on a chair in the floor" code it as 4.2.1but do not code it as 4.6.4 |
| --- |

| **Hazards (includes Surface Contamination and Fall Hazards) (4)** | **Description** |
| --- | --- |
| Surface Contamination | Surface contamination has to result in slip and fall |
| 4.1.1 Liquid/water | Mention/evidence of water or some other forms of liquid. Includes cleaning products, soapy water, beverages, rain, condensation, moisture, freshly mopped, watering plants, in shower, in bathtub, etc. |
| 4.1.2 Grease/Oil | Mention of greasy or oily substance causing slip and fall. (e.g. vegetable oil, engine oil, soap, detergents, shampoo, etc,) |
| 4.1.3 Ice/snow | Mention of ice/snow causing slip and fall. Skiing, snowboarding, ice skating are not coded here. |
| 4.1.4 Food | Mention of food (fruit/vegetables/grains)/rotten food/peels of fruits causing slip and fall |
| 4.1.5 Bodily Fluid | Bodily fluid such as blood, blood products, urine, etc. produced from human or animal bodies |
| Stairs/Steps/Escalator/Elevator |  |
| 4.2.1 Stairs/Steps | Falls on stairs or while working on stairs or steps |
| 4.2.2 Escalator | Falls on escalator or while working on escalator |
| 4.2.3 Elevator | Falls on elevator or while working on elevator, or down elevator shaft |
| 4.3 Ramps | Falls on ramps |
| Elevation equipment/structure/machine/ vehicle |  |
| 4.4.1 Ladder | Falls from or related to ladder |
| 4.4.2 Scaffold | Falls from or related to scaffold |
| 4.4.3 Mechanical aerial lift (e.g. boom lift, scissor lift, fork lift, etc.) | Falls from or related to mechanical aerial lift, bucket of bucket truck |
| 4.4.4 Raised platform/porch/deck | Platform/deck/porch. e.g. Jumping into a pool, falling off a porch, etc |
| 4.4.5 skylight/roof/trees | skylight/roof/trees |
| 4.4.9 Equipment/machinery/vehicle | Falls from other equipment, machinery, or vehicles. e.g. getting in or out of vehicle, falling while standing on a running board, etc. |
| Uneven Ground or Surface | Trips/falls related to uneven ground |
| 4.5.1 cracked/uneven pavement or sidewalk | Trips/falls related to cracked/uneven pavement or sidewalk |
| 4.5.2 Hole/pot hole | e.g. hole in a roof or hole in a ground that already existed before trip and/or fall |
| 4.5.9 Other Uneven ground | Trips related to other uneven grounds such as speed bump, sloping ground, etc. (Exclude skiing) |
| Large Object | Objects that cannot be easily lifted up by an adult |
| 4.6.1 Construction equipment and/or material | Examples: pile of rebar, large sack of cement, equipment, jersey barrier |
| 4.6.2 Construction debris | Construction debris or building materials or byproducts from construction that would otherwise need to be removed as trash or refuse |
| 4.6.3 Packages/products | e.g. large boxes, packages unexpectedly encountered by faller or faller trying to carry/lift |
| 4.6.4 chair/bed/other furniture | Chair/bed/stool/lawn chair or other furniture that can cause a person to slip/trip/fall or that a person falls from |
| 4.6.5 bath tub/shower | Fall occurring while the person is in the bathtub/shower or is stepping over it |
| 4.6.6 toilet/potty chair | Fall occurring while the person is on or attempting to sit on or getting off the toilet/potty chair |
| 4.6.7 Cable, wire, rope, hose | Cable, wire, rope, hose or other entangling material |
| 4.6.9 Other large objects | Other large objects that cannot be lifted easily by an adult. |
| Small Objects | Objects that can be easily lifted up by an adult |
| 4.7.1 Small object clutter | Small objects which are not trash or refuse. Examples, shoes lying on the floor, straps on the floor, clothes, toys, etc. that can cause trip, slip and/or fall |
| 4.7.2 Small object debris | Small objects which are trash or refuse, sand, dust, powder |
| 4.7.3 Small objects (other) | Small objects that are neither clutter nor debris. e.g. objects that are properly placed (trash can placed properly on the floor) |
| 4.8 Curbs/car stops | Includes all trips and/or fall due to curbs/car stops |
| 4.9 Rug/mat/carpet runner | Mention of rug, mat, carpet runner |
| Small Children and Pets/insects |  |
| 4.10.1 Small Children | Any mention of small children |
| 4.10.2 Animal/pets/insects | Any mention of pets/insects |
| 4.11 Footwear | Any mention of footwear. E.g. shoe broke, heels broke |
| 4.12 Slippery surface without mention of any surface contamination | Specific mention of "Slippery surface" or evidence of slippery surface (e.g. waxed floor) without mention of any surface contamination and it does not fall into any of the category above |
| 4.13 Walking/Working/Moving/Standing without any assistive device | Not using any assistive device when "supposed to" has to be specifically mentioned in the text |
| 4.14 none/nothing | specifically mentions that person slipped/tripped over/on NOTHING |
| 4.98 Other/Unknown (default) |  |

**DIRECTION of FALL**

| **Direction of Fall (5)** | **Description** |
| --- | --- |
| 5.1 Forward | Trip up stairs, forwards trip while walking (backward trip is not specifically mentioned), backward slip, falling forward. If it is not explicitly mentioned it can be inferred from the point of impact. For example, if the person hits the face after falling then the direction will be coded "9.1 Forward". Other body parts involved to be qualified for this category include chest, forehead, and nose, both hands injured, both knees injured. However, there has to be evidence of fall. |
| 5.2 Sideways/lateral | Falling sideways has to be explicitly mentioned. There has to be evidence of fall. (e.g. impact on hip, shoulder (either right or left and not both). Broken shoulder alone does not mean the person fell sideways. |
| 5.3 Backward | Falling backward, slipped while going down stairs. If it is not explicitly mentioned it can be inferred from the point of impact. For example, if the person hits the back after slipping/falling then the direction will be coded "9.3 Backward". Other body parts involved to be qualified for this category include back of head, spinal injury, buttocks. However, there has to be evidence of fall. |
| 5.98 Other/Unknown (default) | Other/unknown about the direction of falls that is not categorized into any of the above categories |

**CONTRIBUTING FACTORS**

Select all that apply. Separate by a comma and a space

| **Contributing Factor(s) (6)** | **Description** |
| --- | --- |
| Enter/arrive or Exit/leave building |  |
| 6.1.1 Enter/arrive at building | Applies specifically to a description of a person falling while entering a building or arriving at a building. Doorway has to be mentioned or inferred from the text (door mat is mentioned). |
| 6.1.2 Exit/leave building | Applies specifically to a description of a person falling while exiting or leaving a building. Doorway has to be mentioned or inferred from the text (door mat is mentioned). |
| 6.1.9 unknown (exit/enter not specifically mentioned) - at entrance/doorway | Exiting/entering not specifically mentioned. Doorway has to be mentioned or inferred from the text (door mat is mentioned). |
| Evidence of distraction/not paying attention/unfamiliar environment | Should be used where there is description of fall due to distraction, not paying attention |
| 6.3.1 Evidence of distraction/Not paying attention | There is an explicit indication of evidence of distraction or not paying attention. (e.g. not seeing a curb, unseen objects, using cell phone, reading, etc.) |
| 6.3.2 Unfamiliar environment/surrounding | Unfamiliar with surroundings or where person is explicitly new to a location |
| Physical Vulnerability |  |
| 6.4.1 Pregnant | Pregnancy has to be specifically mentioned in the text. |
| 6.4.2 Elderly/frail | Elderly/frail has to be specifically mentioned in the text |
| 6.4.3 Use of walker, cane, crutches, wheelchair or other assistive devices | Falls occurring when there is use of walker, cane, crutches, or other assistive device (wheelchair) |
| 6.4.4 mention of child, children, baby, kids | Any mention of child or kids |
| 6.4.5 Alcohol use/ intoxication | Alcohol use or drug intoxication (not including prescribed medicine) |
| 6.4.6 Medication | Prescribed/unprescribed medicine |
| 6.4.7 Medical Conditions (including chronic dizziness, BP, Stroke, multiple sclerosis, Parkinson’s disease, etc.) | Any medical conditions mentioned in the text that are usually chronic in nature (e.g. Diabetes, Stroke, Loss of vision, etc.) |
| 6.4.9 Other physical vulnerability | Other preexisting physical vulnerabilities include but are not limited to falls related to ankle/knee gave out, mention of muscle weakness, mention of fever, sporadic/acute dizziness, mention of passing out without any mention of chronic medical condition. Sprained ankle is a diagnosis not a contributing factor. Turning ankle does not necessarily mean that it is a preexisting condition of physical vulnerability. But if the respondent says "I frequently turn my ankle and fall" then it is a sign of pre-existing condition of muscle weakness/balance. |
| 6.5 Dim/poor lighting | Lack of visibility (not related to medical condition) due to dim or poor lighting |
| 6.6 Wind | Requires description of wind causing person to fall |
| 6.98 Other/Unknown (default) | Other or unknown (default) |
|  | |

**LEVEL of FALL**

| **Level (7)** | **Description** |
| --- | --- |
| 7.1 Same Level (default) | Same level is always assumed unless there is evidence to the contrary; Should be used if the person is not able to maintain an upright position and the person slips/trips and/or falls at the same level before the event occurred. (e.g. slipped on floor, tripped on curb, sidewalk). Falls from chairs (unless standing on them) are same level falls. Fall on or up stairs, potholes, curbs, slip on ice getting out of vehicle/truck, jumping on same level, snowboarding, skiing (unless otherwise mentioned) are all coded as "7.1 Same level" falls. Fell on an elevated surface such as porch will be fall at "7.1 Same Level" but fell off or from an elevated surface will be "7.2 To Lower Level" |
| 7.2 To lower level | Should be used if the motion of the fall is generated by gravity due to which the person is not able to maintain an upright position and the person slips/trips and/or falls at the lower level before the event occurred. (e.g. falling down the stairs, ladder, scaffold, bed, or other elevated surface, jumping into the pool), storm drains, ditches, sewers, man-hole, out of a truck if feet not on ground yet. |
